# Pivotal role of the endoplasmic reticulum stress-related XBP1s/miR-22/SIRT1 axis in acute myeloid leukemia apoptosis and response to chemotherapy

**DOI:** 10.1038/s41375-024-02321-8

**Published:** 2024-06-22

**Authors:** Céline Philippe, Manon Jaud, Kelly Féral, Alexandre Gay, Loïc Van Den Berghe, Manon Farce, Marina Bousquet, Stéphane Pyronnet, Laurent Mazzolini, Kevin Rouault-Pierre, Christian Touriol

**Affiliations:** 1 Barts Cancer Institute, Queen Mary University of London, London, UK; 2 Department of Leukemia, Division of Cancer Medicine, The University of Texas MD Anderson Cancer Center, Houston, TX USA; 3 Centre de Recherches en Cancérologie de Toulouse (CRCT), INSERM UMR-1037, CNRS UMR-5071, Université de Toulouse, Toulouse, France; 4 Vectorology Platform, CRCT INSERM UMR-1037 Technological Pole, F-31037 Toulouse, France; 5 Flow Cytometry and Cell Sorting Platform, CRCT INSERM UMR-1037 Technological Pole, F-31037 Toulouse, France

**Keywords:** Acute myeloid leukaemia, Apoptosis

## Abstract

Malignant growth relies on rapid protein synthesis frequently leading to endoplasmic reticulum (ER) overload and accumulation of unfolded or misfolded protein in this cellular compartment. In the ER, protein homeostasis is finely regulated by a mechanism called the unfolded protein response (UPR), involving the activation of signalization pathways mediated by three transmembrane proteins, namely PERK, IRE1 and ATF6. IRE1 endoribonuclease activation leads in particular to the splicing of the cytosolic mRNA encoding the key UPR-specific transcription factor XBP1s. Our study shows that sustained activation of XBP1s expression in acute myeloid leukemia (AML) cells induces apoptosis in vitro and in vivo, whereas a moderate XBP1s expression sensitizes cells to chemotherapeutic treatments. ChIP-seq experiments identified specific XBP1s target genes including the *MIR22HG* lncRNA, the precursor transcript of microRNA-22-3p. miR-22-3p upregulation by XBP1s or forced expression of miR-22 significantly decreases cell’s viability and sensitizes leukemic cells to chemotherapy. We found that miR-22-3p intracellular effects result at least partially from the targeting of the mRNA encoding the deacetylase sirtuin-1 (SIRT1), a well-established pro-survival factor. Therefore, this novel XBP1s/miR-22/SIRT1 axis identified could play a pivotal role in the proliferation and chemotherapeutic response of leukemic cells.

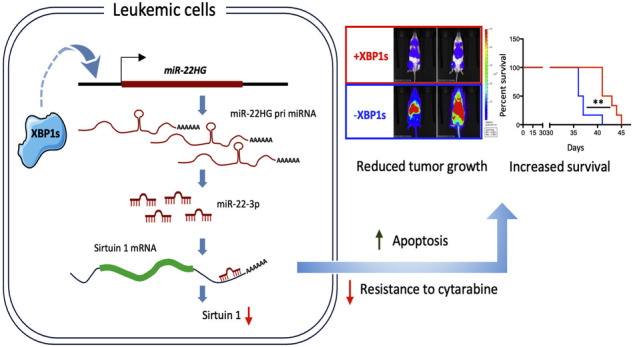

## Introduction

Cancer development relies on a sustained protein synthesis machinery and therefore cancer cells adapt protein quality controls to meet the high demand for protein synthesis [1]. Most cellular stresses (hypoxia, nutrient deprivation, oxidative stress, acidosis, etc.) disrupt endoplasmic reticulum (ER) functions, causing misfolded protein accumulation, which activates the unfolded protein response (UPR) intended to restore ER homeostasis [2–5]. However, if the stress is not resolved, UPR can also lead to cell death [3]. UPR signaling involves the activation of three ER transmembrane proteins (i) the kinase PERK (protein kinase R (PKR)-like ER kinase) (ii) the bifunctional kinase/endoribonuclease IRE1 (inositol requiring enzyme 1), and (iii) the transcription factor ATF6 (activating transcription factor 6) [2, 6, 7].

IRE1, the most conserved arm of UPR, possesses two cytoplasmic catalytic domains (i) a protein kinase domain and (ii) an endoribonuclease domain [2, 8, 9]. Upon activation, the RNAse domain targets specific cytoplasmic mRNA and microRNAs, leading to their decay by the regulated IRE1-dependent decay (RIDD) pathway [10, 11]. In parallel, IRE1α endoribonuclease activity removes a 26 nucleotides sequence from X-box binding protein 1 (*XBP1*) mRNA coding sequence, inducing a translational frameshift prior re-ligation by the tRNA ligase RTCB [12–14]. This “spliced” mRNA is translated into a 376-amino-acids-long isoform XBP1s (s for “spliced”), coding for a potent transcription factor, which regulates stress response genes [15–17], including genes involved in protein folding, trafficking, and also some components of the ER-associated protein degradation machinery.

XBP1s expression in solid tumors is frequently associated with a poor prognosis, while in lymphoma and leukemia its role is controversial [18]. For instance, impaired XBP1 activation is a hallmark of one major germinal center B cell-like diffuse large B cell lymphoma subtype and contributes to tumor growth [19]. In acute myeloid leukemia (AML), XBP1s expression correlates with a favorable outcome upon cytarabine and etoposide therapy [20, 21]. Conversely, IRE1 has also been described to drive pro-survival signals in AML and pre-leukemic stem cells [22, 23].

In order to decipher the role of XBP1s in AML, we generated a model enabling conditional expression of the XBP1s isoform in six different cell line models. Our results demonstrate that sustained XBP1s expression can activate the apoptotic-signaling pathway both in vivo and in vitro. Interestingly, a lower and non-toxic XBP1s expression level sensitizes chemoresistant-AML-cell line OCI-AML3 to cytarabine (aracytine) treatment. By integrating RNA and ChIP-sequencing data, we revealed an XBP1s-dependent upregulation of the long noncoding RNA MIR22HG, precursor of miR-22. Interestingly, mature miR-22 is downregulated in AML patient cells [24] and represses genes involved in the DNA damage response [25, 26]. Here we demonstrate that miR-22 mediates apoptosis and enhances the efficacy of aracytine treatment in AML cells via sirtuin-1 (SIRT1) translational inhibition. Taken together these results identify a novel ER stress-induced axis: XBP1/miR-22/SIRT1, as an effector of apoptosis and chemosensitivity in AML.

## Materials and methods

### Generation of inducible cell lines and shRNA-mediated gene knockdown

The construction of lentiviral vectors expressing the XBP1s, XBP1 shRNAs and miR-22 constructs as well as transductions and establishment of stable and inducible cell lines are described in the Supplementary Information.

### Cell culture and treatments

All leukemic cell lines were obtained from the Leibniz Institute DSMZ or ATCC and grown as recommended by the provider. The clinical and mutational features of our 6 AML cell lines are described in Supplementary Table 1. All drugs used were purchased from Sigma-Aldrich.

### Reverse transcription and quantitative PCR

RNA was extracted as previously described [27]. For mRNA and long noncoding RNA, 500 ng of total RNA were reverse transcribed using the PrimeScript^TM^ RT-PCR Kit (Clontech) according to the manufacturer’s protocol. Reverse transcription (RT) reactions were diluted 20-fold and amplification was performed in a total volume of 10 µL containing 5 µL of the SYBR TB Green® Premix Ex TaqII™ from TakaraBio, 1 µL of both primers (final concentration of 300 nM each), and 2 µL of diluted cDNA. qPCR was performed on the StepOnePlus real-time PCR system (Applied Biosystems) and results were analyzed with the StepOne software. For mature microRNA quantification, RT was performed using the miRCURY™ LNA Universal RT kit and qPCR by the miRCURY^TM^ LNA miRNA PCR system (both kits from Qiagen). hsa-let-7a-5p expression was used for normalization. All primers used in this study are listed in Supplementary Table 2.

### Western blotting

Western blots were performed as previously described [28]. The list of the commercial antibodies is given in the Supplementary Methods together with a description of sample preparation and immunodetection processes.

### siRNA and microRNA transfections

*XBP1s* siRNAs (Smart Pool, Dharmacon), si*SIRT1* #1 (s23771, Thermo Fisher) si*SIRT1* #2 (s23770, Thermo Fisher) as well as negative control siRNA were transfected in OCI-AML3 at a final concentration of 10 nM or 50 nM using the Lipofectamine RNAiMAX transfection reagent (Thermo Fisher) according to the manufacturer’s instructions. mirVana^TM^ miRNA mimic hsa-miR-22*-3p* and negative control (Thermo Fisher) were transfected using the same protocol. Cells were harvested 24 or 48 h later for protein and RNA analyses.

### Flow cytometry measurements of apoptosis and cell viability assays

Analysis of apoptosis was done using Annexin V (Annexin-Pacific Blue^TM^) and propidium iodide (PI) (Biolegend # 640928) staining, according to manufacturer’s recommendations and followed by flow cytometry analysis using a MACSQuant® VYB from Miltenyi Biotec. Results were analyzed using the FlowJo^TM^ software.

### Murine xenograft models

All animal procedures were conducted in compliance with the Ethics Committee pursuant to European legislation translated into French Law as Décret 2013‐118 dated 1st February 2013 (APAFIS 3601‐2015121622062840). Animals were used in accordance to a protocol reviewed and approved by the Institutional Animal Care and Use Committee of Région Occitanie (France). All the experiments were carried out in accordance with relevant guidelines and regulations. NSG and nude mice were obtained from Charles River. Mice treatments, tumor engraftment and tumor growth monitoring are detailed in the Supplementary Methods section.

### Chromatin immunoprecipitation (ChIP)

XBP1s expression was induced with 10 ng/mL of doxycycline for 48 h. ChIP was then performed using the ChIP-IT® Express kit (Active Motif) with minor modifications. Additional reagents used and procedures are given in the Supplementary Methods section.

### AML patient samples

AML and normal bone marrow samples were obtained from patients at the Department of Hematology (CHU, Toulouse, France) after consent, in accordance with the Declaration of Helsinki.

### Biotin pull-down assay

Biotinylated mimic miR-22 (purchased from Exiqon) was transfected in 5.10^6^ OCI-AML3 cells using RNAiMAX (Thermo Fisher) at 10 nM final concentration for 24 h. Cells were then harvested and processed for pull-down assay as detailed in the Supplementary Methods section.

### Statistical analyses

Results are presented as mean values ± standard deviations (SD). Differences between two groups were examined using a two-tailed Student’s *t*-test. Survival analyses were performed using the log-rank test. For the subcutaneous xenografts, determination of statistical significance was performed using two-way ANOVA followed by the Bonferroni test. All analyses were performed using GraphPad Prism version 8.4.

## Results

### XBP1s-inducible expression elicits an ER stress-like response and induces apoptosis in vitro

**Fig. 1:**
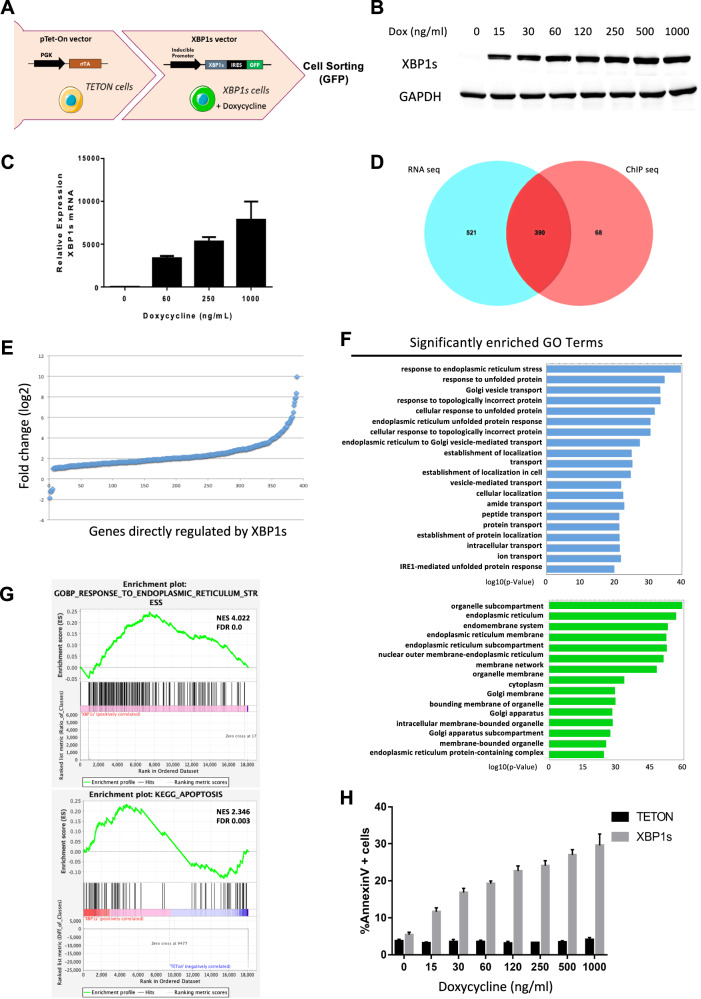
XBP1s expression induces apoptosis in vitro. **A** Schematic of XBP1s-inducible model generation: OCI-AML3 cells were first transduced by a lentivirus expressing the rtTA doxycycline-inducible transactivator. These cells were used as “Tet-On” control cells in the following experiments. These Tet-On cells were then transduced with a lentivector expressing the XBP1s transgene followed by an IRES-eGFP cassette, under the control of a TET-inducible promoter. GFP-positive cells were sorted using flow cytometry after 24 h of doxycycline treatment. OCI-AML3 cells were treated with increasing amounts of doxycycline during 48 h. **B** XBP1s protein level was evaluated by western blot; GAPDH was used as loading control. **C** *XBP1s* expression was evaluated by RT-qPCR. Expression values were normalized to the housekeeping genes *HPRT, MLN51* and *ABL*, and are depicted as a ratio of mRNA expression in doxycycline-induced cells relative to untreated cells. **D** Venn diagrams showing the overlap between RNA seq and ChIP seq experiments in OCI-AML3 XBP1s cells treated with 10 ng/ml of doxycycline for 48 h. The 911 genes selected in RNA seq are the ones with expression increased or inhibited by more than twofold in XBP1s cells compared to control cells. The 458 genes, shortlisted from the ChIP seq analysis, display an XBP1s peak at least at 750 bp upstream or downstream of the characterized transcription start site. **E** Variations in the expression level of the 390 genes showing modulated expression in OCI-AML3-XBP1s cells upon treatment with doxycycline and overlapping between RNA seq and ChIP seq experiments. **F** Gene ontology analysis was performed with the 390 genes up- and downregulated overlapping between RNA seq and ChIP seq experiments in OCI-AML3-XBP1s cells treated with doxycycline. **G** Gene set enrichment analysis (GSEA) was performed on the RNA seq full data of OCI-AML3-XBP1s cells treated with doxycycline. NES normalized enrichment score, FDR false discovery rate. **H** Apoptosis levels of OCI-AML3 Tet-On or OCI-AML3-XBP1s cells treated with increasing amounts of doxycycline during 48 h were measured by flow cytometry using Annexin V/PI staining. Data represent mean ± SD (*n* = 3).

To identify the role of IRE1/XBP1 pathway activation in AML, we established an inducible OCI-AML3 cell line with doxycycline-controlled XBP1s expression. Tet-On cells expressing the rtTA transactivator were transduced with a bicistronic lentivector enabling simultaneous expression of XBP1s and the reporter eGFP (Fig. 1A). To validate our model, control cells (Tet-On) or XBP1s cells were treated with increasing amounts of doxycycline, resulting in increased XBP1s expression both at the protein (Fig. 1B) and mRNA level (Fig. 1C).

We next confirmed the functionality of the exogenously expressed XBP1s protein after induction with low doses of doxycycline by analyzing the expression of *DNAJB9* (*ERdj4*), and HSPA5 (BiP), which are highly induced in response to IRE1/XBP1 signaling activation [29] (Supplementary Fig. 1A, B). Moreover, the expression of *DNAJB9* at the mRNA level increased proportionally to XBP1s (Supplementary Fig. 1C). In addition, XBP1s expression has no effect on eIF2α phosphorylation (Supplementary Fig. 1D) indicating that the PERK pathway is not activated upon XBP1s overexpression.

Post-UPR activation, XBP1s mainly regulates stress response genes [30–32]. To address the role of XBP1s, independently of the two other UPR pathways, we performed several analyses comparing XBP1s OCI-AML3 to Tet-On OCI-AML3 treated with 10 ng/mL of doxycycline: (i) ChIP followed by sequencing to determine the XBP1s genome-wide binding pattern, (ii) global RNA sequencing and (iii) micro-RNA sequencing. We cross-referenced RNA sequencing and ChIP sequencing data to identify XBP1s direct targets. We captured 911 mRNAs that were up or downregulated at least by twofold following XBP1s expression (Supplementary Table 3) along with 458 Bio-ChIP-seq peaks located at <750 bp from a characterized +1 transcription start site. We observed that more than 85% of XBP1s peaks (390 genes) overlapped with genes deregulated after XBP1s induction (Fig. 1D and Supplementary Table 4). Among these 390 genes, only 6 were downregulated (≤3.5-fold), while the 384 others were upregulated, including some of them more than 100-fold compared to the control condition (Fig. 1E). These results demonstrate that XBP1s, when activated independently of other UPR pathways, acts essentially as an activating factor. As expected, the 390 differentially expressed genes are mainly involved in response to unfolded protein accumulation, vesicle-mediated transport and are closely linked to the ER- and IRE1-mediated UPR (Fig. 1F). Notably, XBP1s directly upregulated numerous genes associated with protein processing in the ER (Supplementary Fig. 2A). In agreement, gene set enrichment analysis (GSEA) showed that genes associated with response to ER stress and unfolded protein were enriched in our model (Fig. 1G top and Supplementary Fig. 2B). Moreover, hallmark gene set enrichment analysis also revealed an upregulation of genes involved in apoptosis (Fig. 1G, bottom). Thus, we examined whether XBP1s expression could affect OCI-AML3 cell viability. Flow cytometry analysis demonstrated that increased expression of XBP1s triggers apoptosis in a doxycycline dose-dependent way while doxycycline had no effect on apoptosis in Tet-On control cells (Fig. 1H).

To further validate apoptosis mediated by XBP1s expression observed in OCI-AML3 in a wider panel of AML cell lines, we established five additional tetracycline-inducible cell lines expressing the XBP1 spliced isoform: OCI-AML2, MOLM-14, MV4-11, THP1 and HL60 (Supplementary Fig. 3A). Inducible XBP1s transgene expression was validated both at the mRNA and protein level (Supplementary Fig. 3B–F) and by analyzing the expression levels of downstream targets, i.e., DNAJB9 and HSPA5 (Supplementary Fig. 4). Flow cytometry analyses confirmed that apoptosis correlates XBP1s’ expression in a doxycycline dose-dependent manner in all cell lines while doxycycline had no effect on apoptosis in Tet-On control cells (Supplementary Fig. 3B–F). These results were further validated with cleaved-PARP detection in cells expressing XBP1s (Supplementary Fig. 5).

### Increased XBP1s expression impairs tumoral progression and induces apoptosis in vivo

**Fig. 2:**
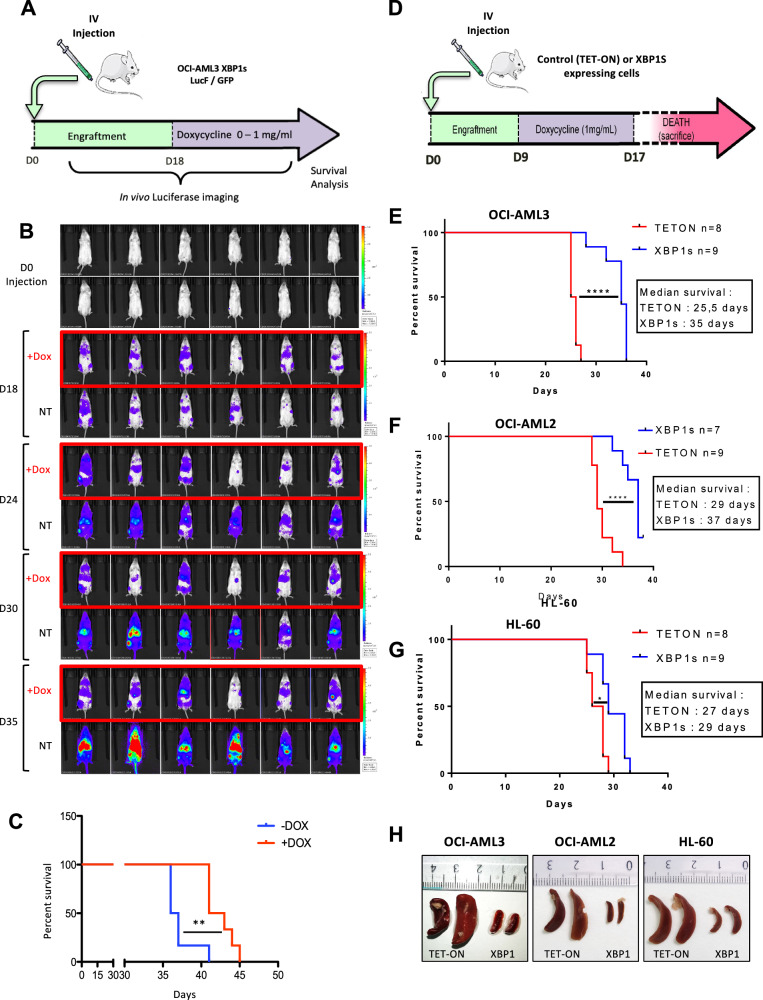
Transient XBP1s expression slows tumor growth in vivo and extends mice survival. **A** NSG mice (*n* = 6 for each group) were injected intravenously with OCI-AML3-XBP1s LucF/GFP-expressing cells. Eighteen days after engraftment, when the luciferase signal was detectable in all the animals, doxycycline was added (or not) at 1 mg/ml in the drinking water. **B** Leukemia development in mice was monitored by bioluminescence imaging with the IVIS Spectrum CT System (PerkinElmer) at the indicated time points. **C** Survival analysis of each group of mice was performed using a log-rank test (**p ≤ 0.01). **D** NSG mice were intravenously injected with either XBP1s-inducible AML cells or their control (Tet-On) counterparts. After a 9 days engraftment, doxycycline was added at the concentration of 1 mg/mL in drinking water during 8 days. **E**–**G** Survival analyses were then performed using the log-rank test (*p ≤ 0.05; ****p ≤ 0.0001) **E** injection of OCI-AML3 cells **F** injection of OCI-AML2 cells **G** injection of HL60 cells. **H** Analysis of spleen size after euthanasia of mice in each of the control (TET-ON) or XBP1s-expressing cell models.

To assess the impact of chronic XBP1s expression on leukemic progression, we transduced OCI-AML3 cells expressing XBP1s with a lentivector constitutively expressing a firefly luciferase. These cells were then injected intravenously into NSG mice. After 18 days, when the luciferase signal was detectable by bioluminescence imaging in all the animals, drinking water was supplemented (or not) with 1 mg/ml of doxycycline (Fig. 2A). Upon treatment mice exhibited a profound delay in leukemia progression compared to untreated mice (Fig. 2B). Furthermore, overall survival of doxycycline-treated mice was significantly extended compared to untreated control mice (Fig. 2C). In order to evaluate whether a transient activation of XBP1s expression could also inhibit leukemic progression, OCI-AML3-, OCI-AML2- or HL60-XBP1s cells or control Tet-On cells were intravenously injected into NSG mice and, after 9 days of engraftment, drinking water was supplemented with 1 mg/ml of doxycycline for 8 days (Fig. 2D). Transient XBP1s expression significantly increased the overall survival of mice for all tested AML cell lines (Fig. 2E–G). Mice injected with the control Tet-On cells displayed splenomegaly, a feature that was absent in mice injected with the XBP1s-expressing cells (Fig. 2H).

**Fig. 3:**
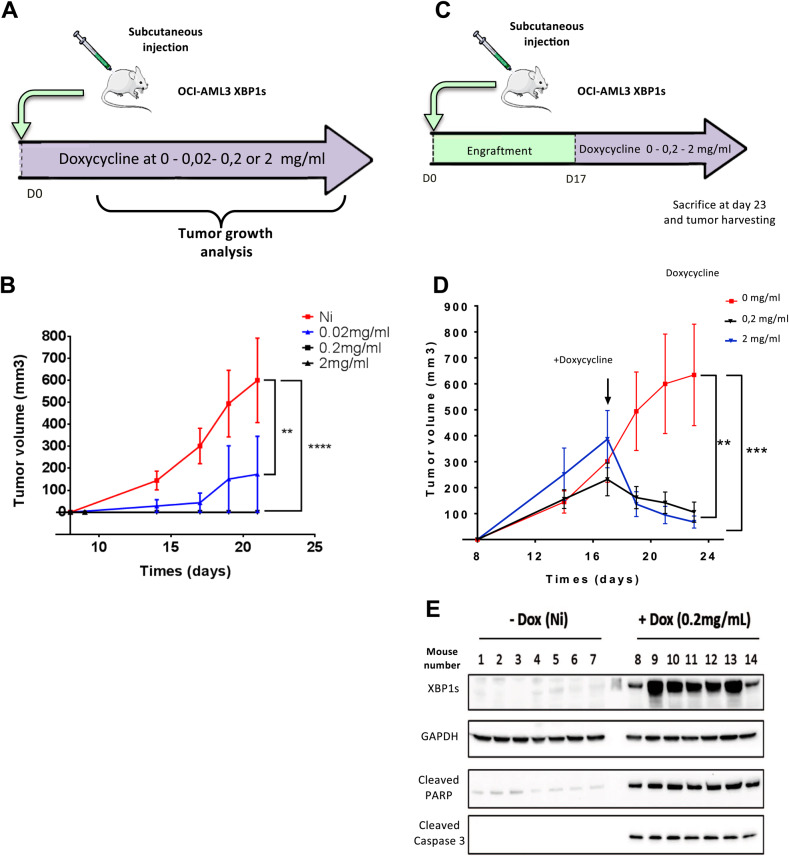
XBP1s expression induces tumor regression and cell apoptosis in vivo. **A** Nude mice were grafted subcutaneously with OCI-AML3 XBP1s-expressing cells and were treated without (Ni) or with doxycycline at 2 mg/ml, 0.2 mg/mL or 0.02 mg/ml. **B** The mean tumor volume in mm^3^ was calculated over a 22-day period. Data represent mean ± SD (*n* = 8 for each group). Statistical analyses were performed by two-way ANOVA with Bonferroni correction (**p ≤ 0.01, ****p ≤ 0.0001). **C** Nude mice were injected subcutaneously with OCI-AML3 XBP1s cells. Seventeen days post injection, mice were exposed (or not), to doxycycline dissolved in their drinking water at a concentration of 2 mg/ml or 0.2 mg/mL. **D** The mean tumor volume in mm^3^ was calculated every 2 days after doxycycline treatment. Data represent mean ± SD (*n* = 7 for each group). Statistical analysis was performed by two-way ANOVA with Bonferroni correction (**p ≤ 0.01, ***p ≤ 0.001). **E** XBP1s expression, PARP and Caspase 3 cleavage were analyzed by western blotting on protein extracts from tumor samples collected at day 23 (end point) and using GAPDH as loading control. Samples 1–7 correspond to individual mice untreated with doxycycline (red curve). Samples 8–14 correspond to individual mice treated with a dose of 0.2 mg/mL of doxycycline in drinking water (black curve).

In order to confirm that chronic activation of XBP1s expression impairs leukemic progression, we used an orthogonal approach where OCI-AML3 cells expressing XBP1s were subcutaneously xenografted in athymic mice (Fig. 3A). While efficient engraftment was observed in mice without doxycycline treatment, supplementation of drinking water with 2 or 0.2 mg/ml of doxycycline after injection completely prevented engraftment. Even after 3 weeks, and at a very low dose of doxycycline (0.02 mg/ml), engraftment was very inefficient (Fig. 3B). To further validate XBP1s anti-tumoral properties, doxycycline was added to the mice’s drinking water 17 days post injection when all tumors have reached an average size of 300 mm^3^ (Fig. 3C). XBP1s induction caused dramatic tumor regression compared to untreated mice (Fig. 3D). At day 23, experiment was stopped and any remaining tumors were collected for analysis of their protein content. Western blot analyses of individual mouse tumor extracts revealed that XBP1s expression was accompanied by the cleavage of both PARP and Caspase 3 (samples 8–14), thus confirming apoptosis-signaling induction in vivo (Fig. 3E). These data further demonstrated XBP1s anti-leukemic activity in vivo.

### Increased XBP1s expression potentiates chemotherapeutic treatment in OCI-AML3 resistant cells

**Fig. 4:**
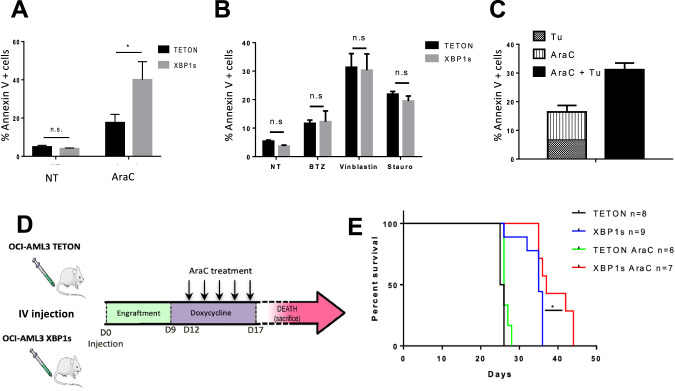
XBP1s expression restores sensitivity to aracytine in the chemoresistant cell line OCI-AML3, both in vitro and in vivo. **A** OCI-AML3 Tet-On (control) and XBP1s received a 24 h treatment of doxycycline at 4 ng/mL, followed by a 24 h treatment with aracytine (10 µM). Percentage of apoptotic cells was measured by flow cytometry using Annexin V/PI staining. **B** OCI-AML3 Tet-On and XBP1s-inducible cells were treated with doxycycline (4 ng/mL) for 24 h and then with bortezomib (BTZ), vinblastine or staurosporine (Stauro), respectively, at 5 nM, 0.5 μM and 0.2 μM for 24 h or left untreated (NT). Data represent mean ± SD (*n* = 3). Statistical analyses were performed using unpaired *t*-tests; *p ≤ 0.05. **C** Apoptosis was assessed by Annexin V/propidium iodide (PI) staining in OCI-AML3 cells. Data represent mean ± SD (*n* = 3). The left side represents the apoptosis induced by tunicamycin and aracytine treatment alone. The right side represents the apoptotic response induced by a combinatorial treatment of the ER stress inducer tunicamycin and aracytine which appears significantly higher than the sum of the separate effects of each individual treatment, indicating that ER stress potentiates aracytine treatment. **D** Schematic of the in vivo procedure. NSG mice were injected (day 0) with 2 million of OCI-AML3 Tet-On (control) and XBP1s cells. After a 9 days engraftment, doxycycline was added at 1 mg/mL in drinking water during 8 days. At day 12, mice were daily injected intraperitoneally with aracytine at 30 mg/kg during 5 days. **E** Survival analyses of the treated mice were performed using a log-rank test (*p ≤ 0.05).

Considering that chronic low XBP1s level in cancer cells supports cell survival, we selected a doxycycline dose that does not trigger cell death (i.e., 4 ng/mL) to investigate the effects of XBP1s’ expression during chemotherapeutic treatment. Annexin V analysis of XBP1s-inducible OCI-AML3 cells by flow cytometry revealed that XBP1s expression sensitized cells to aracytine (Fig. 4A), but not to bortezomib, vinblastine or staurosporine (Fig. 4B) compared to Tet-On control cells. For completeness, we investigated whether ER stress induction by tunicamycin would impact cell survival upon aracytine treatment. Indeed, the combination potentiated apoptosis (Fig. 4C).

To further confirm in vivo the effect of XBP1s on chemosensitization, OCI-AML3 expressing XBP1s and control Tet-On cells were intravenously injected into NSG mice. After 9 days of engraftment, drinking water was supplemented with 1 mg/ml of doxycycline in order to induce XBP1s expression during 8 days. Three days after the beginning of doxycycline treatment, mice were daily injected with aracytine for 5 days (Fig. 4D). With the Tet-On control cells, no survival improvement was observed between untreated and aracytine-treated mice (Fig. 4E) while dox-induced XBP1s expression increased mice survival. Strikingly, XBP1s expression in combination with aracytine treatment further improved mice survival, thus confirming XBP1s-dependent chemosensitization in vivo (Fig. 4E).

### MIR22HG is a direct target of XBP1s and is upregulated by XBP1s upon ER stress

**Fig. 5:**
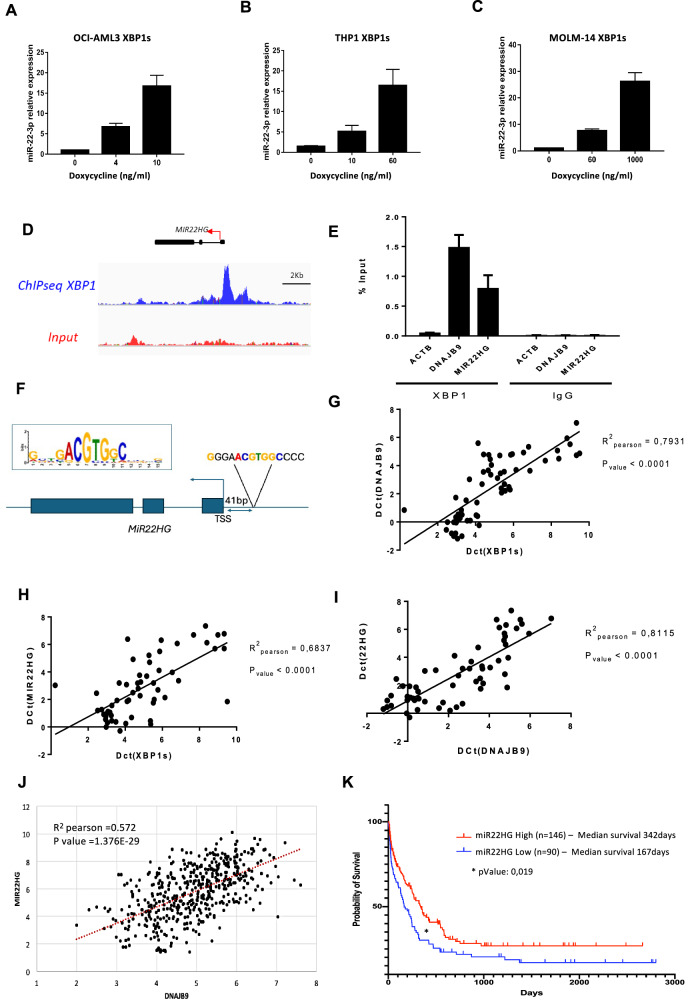
Identification of the MIR22HG lncRNA precursor of mir-22-3p as a direct target of XBP1s. Mature *mir-22-3p* expression levels were assessed by RT-qPCR in OCI-AML3- (**A**), THP1- (**B**) and MOLM-14-XBP1s (**C**) cells treated with doxycycline. Expression values were normalized to the control let-7a microRNA, and are depicted as the relative *mir-22* expression ratio in doxycycline-treated cells compared to untreated cells. Data represent mean ± SD (*n* = 3). **D** Snapshots of ChIP-Seq signals (peaks) representing XBP1s-bound genomic regions in OCI-AML3 cells treated with 10 ng/mL of doxycycline for 48H compared to the input. The MIR22 Host Gene (*MIR22HG*) promoter region and exons are shown. **E** RT-qPCR analysis of *MIR22HG* and *DNAJB9* (positive binding control) promoter regions immunoprecipitated following ChIP assay on OCI-AML3 XBP1s cells treated with 10 ng/mL of Dox and performed using anti-XBP1 or IgG isotype control antibodies. The enrichment of target gene promoter regions is expressed in % of input. The actin B (ACT B) promoter region is used as a negative control. **F** Sequence logos of the consensus *cis*-regulatory elements discovered in XBP1s target promoter, and localization of this consensus sequence in the *MIR22HG* promoter region. Pearson’s correlation analyses showing gene expression levels (quantified by RT-qPCR) of *XBP1s* versus *DNAJB9* (**G**), *XBP1s* versus *MIR22HG* (**H**) and *DNAJB9* versus *MIR22HG* (**I**) in a 55-AML-patient cohort. Expression values are expressed in Delta Ct (D Ct), calculated using housekeeping genes Actin, MLN51, GAPDH and TBP. **J** Correlation of *MIR22HG* and *DNAJB9* expression in 483 AML patient samples from the BEAT AML database. Expression levels are shown on Log2 scales. **K** Survival curves are shown for *MIR22HG* expression in AML using BEAT AML data. To avoid potential bias in data interpretation, we removed here the samples that were not collected at diagnosis but later after the first line of therapy, those presenting with a myelodysplastic syndrome (MDS) or a myeloproliferative neoplasm (MPN) and the samples from patients who could benefit from a transplantation (either bone marrow or cord blood cells engraftment). The Kaplan–Meier curves were finally plotted with such a homogeneous set of *n* = 236 patients. Low and high expression levels of *MIR22HG* are drawn in blue and red, respectively. The *p* value represents the equality of survival curves based on a log-rank test.

Most reported XBP1s targets in the literature are coding genes, with limited information on direct noncoding targets. Among the noncoding targets characterized here, miRnome analysis revealed a substantial upregulation, over 18-fold, of miR-22-3p following XBP1s expression activation (Supplementary Fig. 6A). Upregulation of the mature miR-22-3p microRNA upon XBP1s induction was validated by RT-qPCR in three different AML cell lines (Fig. 5A–C). Interestingly, we also identified *MIR22HG* (miR-22 precursor), as one of the most enriched direct XBP1s long noncoding RNA targets (Supplementary Fig. 6B). The initial ChIP-Seq results (Fig. 5D) were confirmed by ChIP-qPCR experiments which revealed an efficient and specific enrichment of miR22HG promoter region following XBP1 pull-down (Fig. 5E). To uncover potential binding sites for XBP1s in an unbiased manner, we performed de novo searches for over-represented sequences among the promoters bound in our ChIP-on-chip experiments and identified a consensus sequence also found in the *MIR22HG* promoter (Fig. 5F). Gene expression studies using a cohort of 55 primary AML patient samples demonstrated in parallel a positive correlation between *XBP1s*, *MIR22HG* and *XBP1s* known target *DNAJB9* (Fig. 5G–I). The findings from our discovery cohort were confirmed by the validation cohort obtained from the BEAT AML data, showing that there is also a positive correlation between *MIR22HG* and *DNAJB9* expression (Fig. 5J). Altogether these data demonstrate that XBP1s can directly activate transcription of *MIR22HG* and, as a consequence, induce *miR-22* overexpression in AML cells.

Analysis of mir22HG level in samples categorized according to the French–American–British (FAB) classification enabled us to highlight a noticeable progressive increase in miR-22HG expression from M2 to M5 groups (Supplementary Fig. 7). Furthermore, to explore whether *MIR22HG* expression could affect the clinical outcomes, we used the Vizome data analysis tool, which contains data from the BEAT AML cohort. Interestingly, high miR22HG RNA expression was associated with improved overall survival in AML patients (Fig. 5K).

### miR-22 has an anti-leukemic effect in vitro and participates in the XBP1s-induced phenotype

**Fig. 6:**
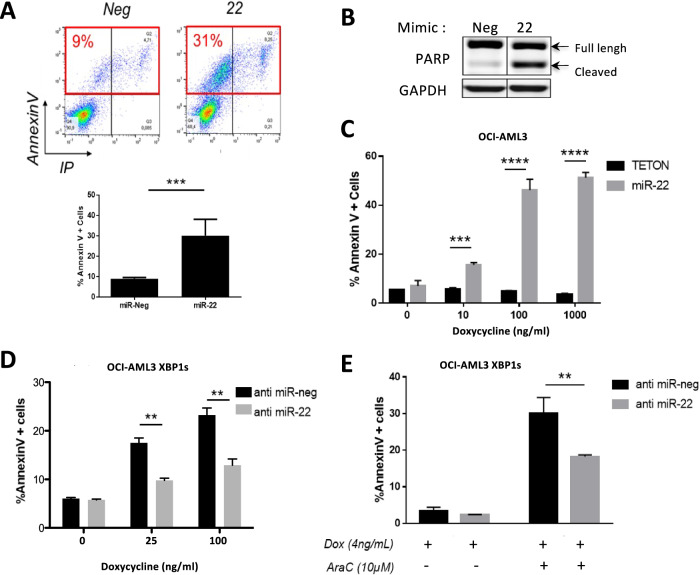
miR-22 recapitulates XBP1s-dependent phenotypes. **A** OCI-AML3 cells were transfected with a *mir-22* mimic (22) or a non-relevant miRNA (Neg) for 48 h. Apoptosis was then assessed by cytometry using Annexin V/PI staining. Lower panel: mean ± SD of three independent experiments. **B** Western blot analysis of cleaved-PARP levels following *mir-22* transfection in OCI-AML3. **C** OCI-AML3 Tet-On cells were transduced with a lentivector expressing a dox-inducible miR-22. Transduced cells and Tet-On control cells were then treated with increasing amounts of doxycycline during 48 h. Apoptosis was measured by flow cytometry using Annexin V/PI staining. Data represent mean ± SD (*n* = 3). **D** OCI-AML3 XBP1s cells were transfected with antisense oligonucleotides against *mir-22* (anti-miR-22) or non-relevant oligonucleotides (anti-miR-neg) as control. After transfection, cells were treated with increasing amounts of doxycycline. After 24 h, apoptosis was quantified. Data represent mean ± SD (*n* = 3). **E** OCI-AML3 XBP1s cells were transfected with antisense oligonucleotides against *mir-22-3p* (anti-miR-22) or non-relevant oligonucleotide (anti-mir-neg) as control. After transfection, cells were treated with 4 ng/mL of doxycycline to induce XBP1s expression and with aracytine (AraC), at 10 μM. Apoptosis was quantified after 24 h. Data represent mean ± SD (*n* = 3). Statistical analyses were performed using unpaired *t*-tests (**p ≤ 0.01, ***p ≤ 0.001, ****p ≤ 0,0001).

To explore the impact of miR-22 expression in cellulo, we transfected OCI-AML3 cells with a miR-22 mimic and observed a significant increase in the apoptotic response compared to the control mimic RNA, as evidenced by the increased number of Annexin V-positive cells as well as increased PARP cleavage (Fig. 6A, B).

To confirm this effect, we generated OCI-AML2 and OCI-AML3 models expressing *miR-22*-*3p* under the control of a doxycycline-inducible promoter. In both cell lines, the addition of increasing doses of doxycycline progressively elicited an apoptotic response in miR-22-expressing cells, but not in Tet-On control cells (Fig. 6C and Supplementary Fig. 8). Moreover, while transfection of an anti-miR-22-3p oligonucleotide had only a slight effect on OCI-AML3 basal apoptosis, it partially rescued XBP1s-induced apoptosis (Fig. 6D) demonstrating that the pro-apoptotic effect of XBP1s is driven at least partially by miR-22-3p expression. Additional experiments revealed that miR-22-3p inhibition through anti-miR-22-3p transfection also significantly decreased aracytine-induced apoptosis when compared to transfection with the negative control (anti-*miR-Neg ;* Fig. 6E), thus confirming that *miR-22* participates in the apoptotic response to aracytine treatment.

### XBP1s elicits apoptotic responses in AML cells in part through miR-22-3p-mediated downregulation of sirtuin-1 deacetylase (SIRT1) expression

**Fig. 7:**
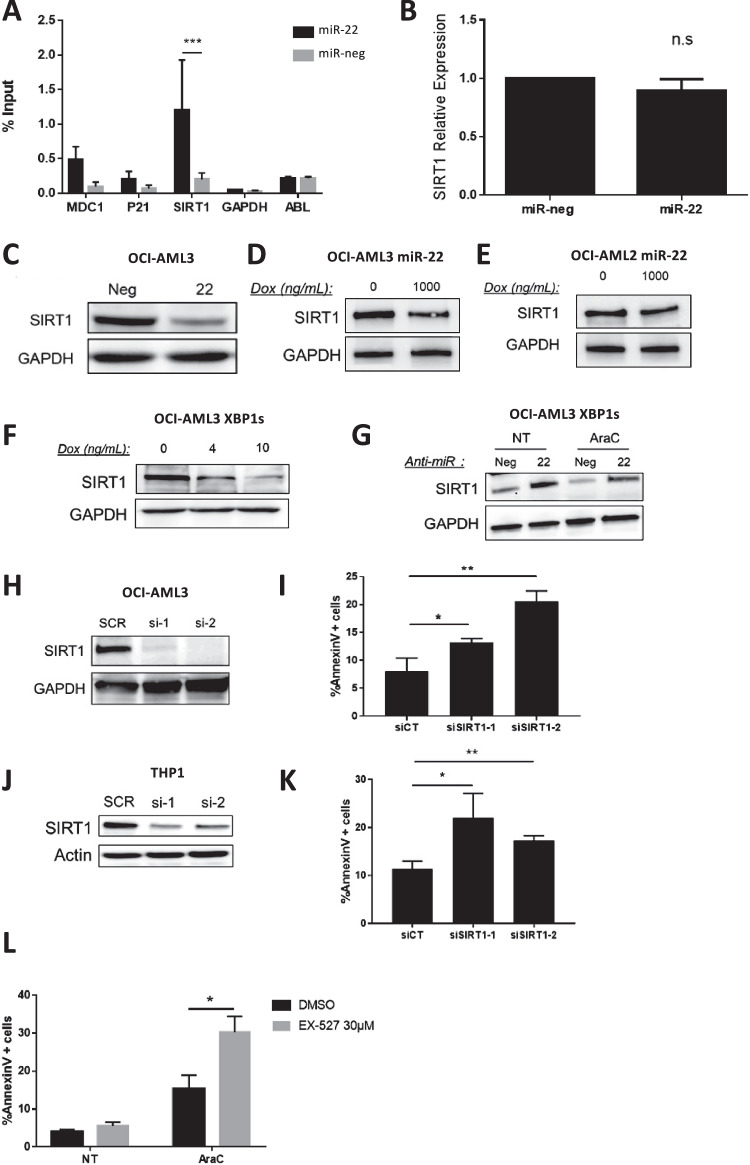
XBP1s/miR-22 axis inhibits SIRT1 expression and compromises leukemic cells survival. **A** RT-qPCR analysis of *MDC1*, *P21*, *SIRT1*, *GAPDH* and *ABL* mRNA levels following an RNA pull-down with a biotinylated *mir-22* mimic transfected in OCI-AML3 wild-type cells. miR-neg: non-relevant pull-down with control miRNA. *GAPDH* and *ABL*: non-target mRNA controls. Data represent mean ± SD (*n* = 3). OCI-AML3 cells were either transfected with a miR-22 mimic (miR-22) or a non-relevant mimic (miR-neg). SIRT1 mRNA and protein levels were determined by RT-qPCR analysis (**B**) and western blotting (**C**), respectively. For RT-qPCR, values were normalized to the expression level of the housekeeping gene *ABL* and are shown as relative *SIRT1* mRNA expression compared to non-relevant mimic transfection. SIRT1 protein levels were determined by western blotting after miR-22 induction in OCI-AML3-miR-22 (**D**) and OCI-AML2-miR-22 (**E**) inducible cells. **F** SIRT1 protein levels were analyzed by western blotting in OCI-AML3 XBP1s-expressing cells treated with 4 ng/mL or 10 ng/mL of doxycycline. **G** SIRT1 protein level was assessed by western blotting in OCI-AML3 XBP1s treated with 4 ng/mL of doxycycline and with (or not) 10 µM aracytine treatment and transfected with an antisense oligonucleotide against miR-22-3P (anti-*miR-22*) or non-relevant oligonucleotide (anti-*miR-neg*). GAPDH was used as a loading control. **H**–**K** OCI-AML3 and THP1 were transfected by two independent siRNAs directed against *SIRT1* (si-1; si-2) or a non-relevant siRNA (siSCR) during 48 h. **H**, **J** siRNA efficiencies were evaluated by western blot using GAPDH or Actin as loading controls. **I**, **K** Apoptosis was measured by flow cytometry using Annexin V/PI staining. Data represent mean ± SD (*n* = 3). **L** OCI-AML3 cells were treated for 96 h with 30 µM of the SIRT1 inhibitor EX-527 or its vehicle DMSO, either with (AraC) or without (NT) aracytine treatment at 10 µM during the last 24 h. Apoptosis was measured by flow cytometry using Annexin V/PI staining. Data represent mean ± SD (*n* = 3). For all the presented experiments statistical analyses were performed using unpaired *t*-tests (*p ≤ 0.05; **p ≤ 0.01, ***p ≤ 0.001).

Some previously described miR-22-3p direct targets, *MDC1*, *P21* and *SIRT1* [33, 34], have been reported to promote cell survival. Using a biotin-labeling-based pull-down procedure we found that *SIRT1* mRNA was efficiently enriched after *miR-22* pull-down in OCI-AML3 cells. (Fig. 7A). Interestingly, the deacetylase SIRT1 was previously found to be frequently overexpressed and considered as an oncogene in AML [35–37]. Whereas no significant reduction of *SIRT1* mRNA was observed after miR-22 mimic transfection (Fig. 7B) SIRT1 protein expression was lowered (Fig. 7C), indicating that miR-22 likely affects SIRT1 expression at the translational level. We confirmed the downregulation of SIRT1 protein expression by miR-22-3p using our two miR-22*-*inducible cell lines (Fig. 7D, E). We also observed a drop of SIRT1 protein levels following dose-dependent induction of XBP1s expression in OCI-AML3 (Fig. 7F) and XBP1s-inducible OCI-AML2, MOLM-14, MV4-11 and THP1 cells (Supplementary Fig. 9). Finally, we showed that anti-miR-22-3p treatment of doxycycline-induced OCI-AML3 XBP1s cells led to the re-expression of SIRT1 protein, both in untreated and aracytine-treated conditions (Fig. 7G). This supports the role of the XBP1s/miR-22-3p axis in downregulating SIRT1.

We then directly downregulated SIRT1 expression in OCI-AML3 and THP1 cells by RNAi. Two independent siRNAs directed against SIRT1 efficiently repressed its expression (Fig. 7H, J) and increased apoptosis (Fig. 7I, K) when compared to controls. Finally, SIRT1 pharmacological inhibition using the highly specific SIRT1 inhibitor EX-527 specifically enhanced the apoptotic response to treatment with aracytine (Fig. 7L). Altogether, these results demonstrated the implication of SIRT1 in the XBP1s-dependent chemosensitization to aracytine.

## Discussion

In tumors, cancer cells are prone to many stresses which can disturb protein synthesis and folding accuracy and overwhelm protein quality control processes thus leading to ER stress and activation of the PERK, ATF6 and IRE1-XBP1s UPR signaling pathways. UPR induction acts initially as a cell protective process aimed to restore protein homeostasis. However, if the damage is excessive, UPR moves from protective-to-cytotoxic in order to eliminate dysfunctional cells [38]. Chemotherapeutic drugs, as strong inducers of stress in cancer cells, could exert their cytotoxic effect at least in part through boosting the transition from protective-to-cytotoxic UPR. Interestingly our recent bibliographic survey on the role played by the UPR in leukemia and the response to treatment with candidate therapeutic molecules revealed that the observed increases in cell death were extremely frequently associated with cytotoxic UPR induction [18], but mechanisms of action have not been explored so far in the vast majority of these studies.

In a large number of cancers, including hematological malignancies [22, 23, 39, 40], activation of IRE1-XBP1s branch was found to initially play an essential safeguard role against apoptosis during cellular stresses. In addition, some previously published functional studies of XBP1s in leukemic cells clearly pointed out a cell protective function [41, 42]. The increased expression of XBP1s, observed in certain cancers is therefore widely considered to primarily act by restoring cell homeostasis in conditions of moderate cellular stress, thus contributing both to promote precancerous development and to increase the resistance of cancer cells to the various stresses they undergo in vivo during tumor development. Blocking the protective IRE1-XBP1s signaling pathway appears therefore as an attractive anti-cancer therapy and the search for pharmacological inhibitors of IRE1 is under active development [43]. However, strong IRE1 activation can also contribute to apoptosis induction [42, 44, 45] by different molecular mechanisms such as hyperactivation of endonuclease activity boosting the RIDD process [42, 44] or activation of the apoptotic JNK signaling pathway [45, 46]. During IRE1-induced pro-apoptotic response, XBP1s expression was found, when tested [41, 42], to retain a cell protective effect which appeared outweighed by the other actions taken by IRE1. However, in lung cancer cells, XBP1s could also switch from a negative to a positive mediator of cytotoxic UPR once cell stress surpasses a specific threshold [47]. This effect was associated with a modification of XBP1’s transcriptional target selectivity allowing the activation of pro-apoptotic genes such as the transcription factor KLF9 [47]. The implication of XBP1s in the induction of pro-apoptotic programs leading either to slowed tumor progression in vivo [19] or to increased sensitivity to Bruton’s kinase tyrosine kinase inhibitor ibrutinib has also been reported in the case of diffuse large B cell lymphomas [48]. Therefore, XBP1s may as well directly contribute to apoptosis induction in certain conditions.

Interestingly, in AML, XBP1 activation has been previously correlated with a favorable outcome upon chemotherapy [21] but the underlying molecular mechanisms remained unknown.

In this study, we aimed to clarify the functions played by XBP1s in leukemic cells. We showed that sustained XBP1s expression induces apoptosis whereas a moderate XBP1s expression, compatible with cell viability, enhances response to aracytine treatment in AML-resistant cells. This last effect is achieved by increasing *XBP1s* expression at RNA level by about tenfold. Interestingly, this increase is of the same order of magnitude as that obtained on endogenous *XBP1* in isolated patient cells treated with tunicamycin, for which activation of *XBP1s* expression ranges from 5- to 25-fold increase (Supplementary Fig. 10). In addition, highly deregulated XBP1s expression, able on its own to induce cytotoxic responses, could presumably also be achieved during treatment with anti-leukemic drugs, most of them having been reported to induce strong UPR response in leukemic cells [18].

We characterized direct XBP1s target genes using ChIP-sequencing, RNA sequencing and microRNA sequencing analyses. From the list of identified targets, we decided to focus our analysis mainly on XBP1s noncoding targets, still poorly characterized so far. We identified 13 annotated long noncoding RNAs as specific XBP1 target genes, including *MIR22HG* (*C17orf91*), the precursor transcript of the microRNA *miR-22-3p* (Supplementary Fig. 6). Interestingly, previous studies demonstrated that *MIR22HG* is expressed in response to hypoxic or chemical stress, two stimuli known to induce ER stress [49, 50]. Here, we showed that induction of MIR22HG expression upon ER stress is XBP1-dependent.

*Mir-22* function and regulation appears very complex [51] and, in leukemia, it has been reported to behave either as oncogenic or anti-tumoral depending on the model system [24, 52, 53].

Here we found that in OCI-AML3 cells miR-22 interacts efficiently with the mRNA of the deacetylase *SIRT1*, a member of the mammalian sirtuin family, and inhibits SIRT1 expression at the translational level, as previously described [34]. SIRT1 was previously reported to be upregulated in AML and identified as an oncogene in this pathology [35, 36]. Consistently, SIRT1 repression in OCI-AML3 and THP1 cells using siRNAs triggered apoptosis (Fig. 7H–K). This clearly demonstrated that this protein acts as a pro-survival factor in AML cells.

SIRT1 is involved in many cellular processes including DNA repair [54] and protects against radiation-induced apoptosis in different diseases such as brain, lung and breast cancers [26, 55, 56]. Moreover, in the latter model, miR-22 overexpression inhibited SIRT1 expression and improved cells response to radiotherapy [26]. In AML, the SIRT1 pharmacological inhibitor Tenovin-6 (Tv-6) was previously shown to potentiate aracytine treatment [35]. Moreover, Tv-6 has also been reported to inhibit SIRT2 and activate p53. Our experiments performed with EX-527, a highly specific inhibitor of SIRT1 [57], confirmed the protective role of SIRT1 against aracytine treatment in AML cells. Conversely, we showed that inhibition of endogenous miR-22 in XBP1s-expressing cells restores SIRT1 expression (Fig. 7G) and partially reverses aracytine-induced apoptosis (Fig. 6E).

Our studies revealed that overexpression of XBP1s contributes to lower the expression level of SIRT1 at least in part through miR-22-3p upregulation and that this process may affect both cell viability and response to cytotoxic drugs. Interestingly, previous studies have shown that SIRT1 may play as well an inhibitory role on the UPR response. Indeed, the activation of SIRT1 in the liver of mice leads to reduced activation of both PERK and IRE1-XBP1s signaling [58]. Moreover, concomitant studies showed that SIRT1 can act as a negative regulator of XBP1s by repressing its transcriptional activity through direct deacetylation [59]. Therefore, a negative feedback loop between SIRT1 and XBP1 may take place in cells to balance their relative activities and finely modulate cell viability.

To conclude, the overall presented data suggest that IRE1/XBP1s signaling may in some conditions act as a tumor suppressor pathway in AML, in particular through activation of the XBP1s/miR-22/SIRT1 axis. Activating this branch of the UPR could therefore represent an attractive additional therapeutic strategy for AML treatment.

### Supplementary information

Supplementary Materials and methods

## Data Availability

The datasets generated during this study are available upon reasonable request to the corresponding author.
