# Supplementary material for: Pivotal role of the endoplasmic reticulum stress-related XBP1s/miR-22/SIRT1 axis in acute myeloid leukemia apoptosis and response to chemotherapy
Source: Leukemia. 2024 Jun 22;38(8):1764–76. doi: 10.1038/s41375-024-02321-8 (PMC11286524; doi:10.1038/s41375-024-02321-8)
Supplement: Supplementary file 1 — Supplementary Materials and methods [file 41375_2024_2321_MOESM1_ESM.pdf]

Supplementary Materials and Methods

Generation of inducible cell lines and shRNA-Mediated Gene Knockdown

The XBP1 spliced isoform coding sequence was PCR amplified with the proofreading Taq polymerase Phusion (Thermofisher) using the primer pair Xba_XBP1_Fw (GGTCTAGA-TAATACGACTCACTATAGGGATGGTGGTGGTGGCAGCCGCGCCG) and XBP1_Sal_Rev (CCGTCGACTGCAGAATTCGAAGCTTGAGCTCGAGTTAGAC-ACTAATCAGCTGGGG). The EMCV IRES GFP cassette was in parallel amplified with the primers pIres2GFP_Sal1_Fw (CAGTCGACGGTACCGCGGGCCCGGGATC-CGCCCCTCTCCCTCCCCCCCCCC) and pIres2GFP_BGL2_REV (TTAGATCTTAC-TTGTACAGCTCGTCCATGCCGAG) using the pIRES2GFP vector from Clontech as a template. These two PCR products were digested respectively by Xba1/Sal1 and Sal1/Bgl2 and ligated into the pTRIPz-TRE-Tight plasmid (Open Biosystem) digested by Xba1/ Bgl2 leading to the fusion of the XBP1s open reading frame to the EMCV IRES GFP sequence downstream of a doxycycline responsive promoter. Constructs were validated by sequencing and used to transduce OCI-AML2-, OCI-AML3-, MV4-11-, MOLM-14-, THP1- and HL-60-TET-On cell lines, first established in the lab by transduction with pTet-On vector (Clontech) which allows the constitutive expression of the rtTA (reverse tetracycline Transactivator) protein. Selection of XBP1s-inducible cells was based on EGFP expression and performed by flow cytometry after treatment with a low dose of doxycycline during 10H. For generation of stable XBP1 knockdown, wild-type cells were transduced with the lentivirus pTRIPZ-XBP1 shRNA (Dharmacon: reference V3THS_387389, Mature Antisense sequence: TCTTCTAAATCTACCACTT). Stably transduced cells expressing the shRNA were selected by treatment with 1 µg/mL of puromycin. miR-22-inducible models were generated using wild-type OCI-AML2 and OCI-AML3 cells stably transduced with shMIMIC Inducible Lentiviral microRNA (Horizon; reference VSH6906-224634676; miRNA sequence: AA-GCUGCCAGUUGAAGAACUGU). All transductions were performed in the presence of retronectin™ reagent, according to the manufacturer's instructions (Clontech).

Protein sample preparation, Western Blotting and immunodetection

After lysis and sonication (10s each separated by a 30s incubation on ice using VCX 130 Ultrasonic Processor (Sonics) at 25% of its power) cellular debris were eliminated by cen-

trifugation (10min at 10,000g, 4°C). The total protein concentration of cell lysates was determined using a Bicinchoninic Acid Assay kit purchased from Sigma-Aldrich. Protein lysates were fractionated on SDS-PAGE 10% and transferred to a nitrocellulose membrane with the Trans-Blot® Turbo™ Transfer System (Bio-Rad). Immunodetections were performed using commercial antibodies against XBP1 (Santa Cruz, sc-7160), phosphorylated eIF2α (Cell Signaling, #9721), total PARP (Cell Signaling, #9532), cleaved PARP (Cell Signaling, #9541), cleaved caspase 3 (Cell Signaling, #9664), SIRT1 (Cell Signaling, #9475), GAPDH (Millipore, MAB374) and Actin (Sigma-Aldrich, a5441) followed by incubation with HRP-conjugated secondary antibodies (Cell Signaling). Protein signals were visualized using the Clarity™ Western ECL reagent from Bio-Rad.

Murine xenograft models and following of tumor growth in vivo

NSG mice were treated by an intraperitoneal injection of busulfan (20 mg/kg) to induce medullar aplasia. 24h after busulfan treatment, mice were intravenously injected with 2 10^6 OCI-AML3, OCI-AML2 or HL-60 XBP1s-inducible cells or Tet-On control cells. At different time points after engraftment, doxycycline was added at 1mg/mL in drinking water and refreshed every 3 days. Mice daily monitoring was performed to detect symptoms of disease (ruffled coat, hunched back, weakness, and reduced motility). For *in-vivo*-chemosensitivity assay, 3 days after doxycycline-treatment onset mice were intraperitoneally injected daily with aracytine at 30mg/kg for 5 days. For subcutaneous xenografts, a total of $2.5.10^6$ OCI-AML3 XBP1s cells were injected into both flanks of nude mice. Mouse body weight and tumor volumes were measured every day. Doxycycline (different concentration) was added in the drinking water and refreshed every 3 days. At the end of the experiment, mice were humanely sacrificed and subcutaneous tumors were harvested and proteins extracted for further analyses.

Chromatin Immunoprecipitation (ChIP)

ChIP was performed using the ChIP-IT® Express kit from Active Motif. In order to cross-link proteins to DNA, cells were treated with 1% formaldehyde for 10min at room temperature, then with glycine according to the manufacturer’s protocol. Chromatin was sheared by sonication into relatively uniform 300pb fragments ("Input" fraction). This chromatin fraction was subsequently immunoprecipitated with two different XBP1 antibodies (Santa Cruz, sc-7160

and Biolegend, 619502), using magnetic beads. IgG isotype (Biolegend, 910801) was used as immunoprecipitation control. DNA was eluted, and then de-crosslinking and purified using phenol-chloroform extraction. The immunopurified fractions obtained with this protocol were then used for ChIP-sequencing and for ChIP-qPCR experiments. For ChIP-qPCR, 1/10 (2µl) of input or immunoprecipitated DNA was analyzed by qPCR with SYBR green (Takara Bio Inc.) on the StepOnePlus real-time PCR system (Applied Biosystems). Results obtained with the immunoprecipitated chromatin fractions are expressed as the percentage of input and represent the mean value of at least three independent ChIP experiments. For each ChIP experiment specificity of the immunoprecipitation was controlled by a parallel ChIP performed with an irrelevant antibody (igG).

RNA-seq et miRnome

Total RNA from OCI-AML3 Tet-ON and XBP1s treated with 4ng or 10 ng of doxycycline for 48h were purified using the Trizol (Ambion) extraction protocol according to the manufacturer's instructions. RNA quantification and purity were determined by using a Clariostar. RNA integrity and quality were determined by using a Fragment Analyzer. Only samples with RNA Integrity Number (RIN) >9 were sent for RNA-Sequencing. Library preparation and sequencing were performed by Eurofins GATC Biotech company. Paired-end, 120 million strand-specific reads of ~125 nucleotides/ sample were generated on an Illumina HiSeqTM2000 for RNA sequencing and 10 million reads of 50 nucleotides /sample were generated for miRnome analysis.

Binding site motifs analysis

Motif analysis was performed following peak calling, using FASTA files as input for the MEME-Chip online tool (meme-suite.org). The program finds motifs centrally enriched and analyses them for similarity. The JASPAR vertebrate's motif database was used as a reference. Default settings were used for this analysis with MEME Motif width between 6 and 30, DREME Motif E-value<0.05, CentriMo motif match score ≥5, and CentriMo E-value threshold <10.

Biotin Pull Down Assay

After a 24h transfection with the biotinylated microRNA-22 mimic the cells were centrifuged and resuspended in an immunoprecipitation (IP)-Buffer (25mM Tris-HCL pH7.4, 200mM NaCl, 0.2% Triton™ X-100, 5mM Mg Acetate, 1mM DTT) supplemented with RNAseOUT (Thermo Fisher) and Complete™ protease inhibitor cocktail (Roche). Samples were sonicated (2 times 10s each separated by a 30s incubation on ice using VCX 130 Ultrasonic

Processor (Sonics) at 25% of its power) and lysates were used for pull down. Pierce™ Magnetic beads from ThermoFisher were washed with IP-buffer and blocked with yeast tRNA and BSA for 1h at 4°C. Beads were washed twice and incubated with the lysate for at least 1h30 at 4°C on an end-to-end rotator. Beads was washed 5 times with IP buffer and RNA extraction was performed using TRIzol® total RNA isolation reagent (Invitrogen), following the manufacturer's protocol. Then, 11µL of immunoprecipitated RNA extract was used for RT with the Superscript III reverse transcription kit (Invitrogen). RT reactions were diluted 10-fold prior to qPCR (see primer list in the Supplementary Table 2).

***Supplementary Table 1**:* Clinical and mutational features of the AML cell lines used in this study

Name	*Age / Gender*	*FAB*		*Karyotype*	*Translocation / Fusion genes*	*FLT3*	*NPM1*	*DNMT3A*	*NRas*	*KRas*	*p53*	*c-Myc*	*Cytarabine resistance*
**HL-60**	36y / F	M2	Dx	Complex	-	wt	wt	wt	mut G61L	wt	Null; deleted	Amplified	Sensitive
**MV4-11**	10y / M	M5	Dx	Complex	t(9;11)(p22;q23) / KMT2A-MLLT3	ITD homozygotous	wt	wt	wt	wt	wt	Overexpressed	Sensitive
**MOLM-14**	20y / M	M5	Relapse	Complex	t(9;11)(p22;q23) / KMT2A-MLLT3	ITD heterozygotous	wt	wt	wt	wt	wt	Overexpressed	Sensitive
**OCI-AML2**	65y / M	M4	Dx	Complex	t(8;21)(q22;q22) / RUNX1-CBFb	wt	wt	mut R635W	wt	wt	wt	-	Sensitive
**OCI-AML3**	57y / M	M4	Dx	Complex	-	wt	mut	mut R882C	mut G61L	wt	wt	-	Resistant
**THP-1**	1y / M	M5	Relapse	Complex	t(9;11)(p22;q23) / KMT2A-MLLT3	wt	wt	wt	mut G12D	wt	mut (Heterozygous)	-	Sensitive

Supplementary Table 2: List of PCR primers used in these studies

GENE	FORWARD PRIMER	REVERSE PRIMER
XBP1s	5'-CTGAGTCCGCAGCAGGTGCAG-3'	5'-ATCCATGGGGAGATGTTCTGG-3'
MIR22HG	5'-CCTCGTGCAGCAACCCC-3'	5'-GTGAGGGCGTGAGAGGAAC-3'
SIRT1	5'-ACCTCCTCATTGTTATTGGGTCT-3'	5'-GGGCACTTCATGGGGTATGG-3'
DNAJB9	5'-AAAATAAGAGCCCGGATGCT-3'	5'-CGCTTCTTGGATCCAGTGTT-3'
MDC1	5'-ATTAGCTGCTGTGGAGGCAC-3'	5'-CCCGTAGTGGAATGGAGCAA-3'
P21	5'-ACTCTCAGGGTCGAAAACGG-3'	5'-GCGGATTAGGGCTTCCTCTT-3'
ABL	5'-TGGAGATAACACTCTAAGCATAAC TAAAGGT-3'	5'-GATGTAGTTGCTTGGGACCCA-3'
HPRT	5'-TGCTTTCCTTGGTCAGGCAGT-3'	5'-CTTCGTGGGGTCCTTTTCACC-3'
TBP	5'-GCCTCCCCCACCCCCTTCTTT-3'	5'-GCCACACCCTGCAACTAACATCC-3'
Actin	5'-CACCATTGGCAATGAGCGGTTC-3'	5'-AGGTCTTTGCGGATGTCCACGT-3'
GAPDH	5'-CAACGACCACTTTGTCAAGCT-3'	5'-CTCTCTTCCTCTTGTGCTCTTGC-3'
**ChIP PRIMERS**		
DNAJB9	5'-GGTTTAAGCACCGCCTTTTCG-3'	5'GTGGAAAACTGTTGTTGCTGCTA-3'
MIR22HG	5'- CCGGCCAATAGACGGACA-3'	5'- CTTTCGCCTGCTCTTTAGGAC-3'
ACTIN	5'-GGGACTATTTGGGGGTGTCT-3'	5'-TCCCATAGGTGAAGGCAAAG-3'

***Supplementary table 3*: XBP1s-regulated genes identified by RNA sequencing**

List of the 911 genes that are regulated more than 2 fold upon XBP1s expression in OCI-AML3 XBP1s cells compared to TETON control cells after treatment with 10ng/mL doxycycline for 48H (Fold changes are expressed in log2).

Ensemble ID	gene name	Fold change (Log2)
ENSG00000110042	DTX4	-3,1438338
ENSG00000139626	ITGB7	-2,391103306
ENSG00000171509	RXFP1	-2,341747309
ENSG00000121807	CCR2	-2,27310699
ENSG00000122025	FLT3	-2,215917379
ENSG00000269243	AC008894.2	-2,05944241
ENSG00000149516	MS4A3	-2,03932995
ENSG00000145703	IQGAP2	-2,018309071
ENSG00000101347	SAMHD1	-1,998848515
ENSG00000198771	RCSD1	-1,985464382
ENSG00000171791	BCL2	-1,969943187
ENSG00000184602	SNN	-1,959571041
ENSG00000183307	TMEM121B	-1,927630915
ENSG00000134986	NREP	-1,860783371
ENSG00000143858	SYT2	-1,848414783
ENSG00000019582	CD74	-1,82921327
ENSG00000196415	PRTN3	-1,792588955
ENSG00000179862	CITED4	-1,772150409
ENSG00000144354	CDCA7	-1,766306815
ENSG00000120280	CXorf21	-1,723061129
ENSG00000183688	RFLNB	-1,699428381
ENSG00000204472	AIF1	-1,683926235
ENSG00000184992	BRI3BP	-1,681992615
ENSG00000139880	CDH24	-1,681943052
ENSG00000140968	IRF8	-1,676929839
ENSG00000158258	CLSTN2	-1,622291914
ENSG00000183741	CBX6	-1,620418252
ENSG00000175602	CCDC85B	-1,618791952
ENSG00000215246	AC116351.1	-1,616383652
ENSG00000145506	NKD2	-1,597234823
ENSG00000136490	LIMD2	-1,596668905
ENSG00000068831	RASGRP2	-1,57904004
ENSG00000143546	S100A8	-1,563189791
ENSG00000101220	C20orf27	-1,546197812
ENSG00000137642	SORL1	-1,542925184
ENSG00000163220	S100A9	-1,513483257
ENSG00000165168	CYBB	-1,492523253
ENSG00000173621	LRFN4	-1,489537918
ENSG00000152518	ZFP36L2	-1,487963486
ENSG00000175130	MARCKSL1	-1,479831277
ENSG00000145569	FAM105A	-1,465128712

ENSG00000102879	CORO1A	-1,454284601
ENSG00000090382	LYZ	-1,442985343
ENSG00000005249	PRKAR2B	-1,390986339
ENSG00000221869	CEBPD	-1,364419601
ENSG00000178773	CPNE7	-1,361673454
ENSG00000126759	CFP	-1,357727658
ENSG00000205213	LGR4	-1,356786865
ENSG00000247982	LINC00926	-1,354164141
ENSG00000255587	RAB44	-1,326991862
ENSG00000125520	SLC2A4RG	-1,325103386
ENSG00000160949	TONSL	-1,324433282
ENSG00000112782	CLIC5	-1,321407516
ENSG00000104870	FCGRT	-1,309626471
ENSG00000133055	MYBPH	-1,302657064
ENSG00000116985	BMP8B	-1,300456571
ENSG00000163013	FBXO41	-1,294262228
ENSG00000059588	TARBP1	-1,293125886
ENSG00000203668	CHML	-1,293038171
ENSG00000163563	MNDA	-1,286231734
ENSG00000115232	ITGA4	-1,271722667
ENSG00000171608	PIK3CD	-1,269553555
ENSG00000130881	LRP3	-1,264437107
ENSG00000101460	MAP1LC3A	-1,261257345
ENSG00000112759	SLC29A1	-1,260676914
ENSG00000128951	DUT	-1,260262172
ENSG00000228716	DHFR	-1,255957747
ENSG00000124813	RUNX2	-1,250825792
ENSG00000180900	SCRIB	-1,248406843
ENSG00000020922	MRE11	-1,244894274
ENSG00000130511	SSBP4	-1,240453575
ENSG00000117632	STMN1	-1,239688826
ENSG00000073111	MCM2	-1,227338847
ENSG00000274536	AL034397.3	-1,225036722
ENSG00000020181	ADGRA2	-1,2216594
ENSG00000205269	TMEM170B	-1,219170741
ENSG00000137309	HMGA1	-1,219123338
ENSG00000177084	POLE	-1,212620714
ENSG00000101057	MYBL2	-1,2105147
ENSG00000188486	H2AFX	-1,210374624
ENSG00000145386	CCNA2	-1,204571272
ENSG00000146083	RNF44	-1,201639358
ENSG00000118513	MYB	-1,200860042

ENSG00000157557	ETS2	-1,199633224
ENSG00000205268	PDE7A	-1,198238627
ENSG00000158473	CD1D	-1,197324157
ENSG00000132600	PRMT7	-1,194032368
ENSG00000178035	IMPDH2	-1,191585061
ENSG00000142185	TRPM2	-1,183281577
ENSG00000164853	UNCX	-1,177702274
ENSG00000132182	NUP210	-1,176482947
ENSG00000131747	TOP2A	-1,166180771
ENSG00000196588	MKL1	-1,163605259
ENSG00000138095	LRPPRC	-1,15198535
ENSG00000132199	ENOSF1	-1,14938987
ENSG00000155629	PIK3AP1	-1,14643576
ENSG00000156802	ATAD2	-1,146176263
ENSG00000112787	FBRSL1	-1,142312778
ENSG00000117724	CENPF	-1,135086441
ENSG00000164024	METAP1	-1,1320228
ENSG00000111252	SH2B3	-1,129917796
ENSG00000132819	RBM38	-1,129009168
ENSG00000204371	EHMT2	-1,125209041
ENSG00000100297	MCM5	-1,124371515
ENSG00000123213	NLN	-1,121530283
ENSG00000125869	LAMP5	-1,118386854
ENSG00000135776	ABCB10	-1,112988945
ENSG00000197561	ELANE	-1,11268347
ENSG00000101444	AHCY	-1,105873237
ENSG00000127586	CHTF18	-1,088303891
ENSG00000140853	NLRC5	-1,0880073
ENSG00000150681	RGS18	-1,087919584
ENSG00000135596	MICAL1	-1,086186038
ENSG00000149499	EML3	-1,085205784
ENSG00000164104	HMGB2	-1,083361566
ENSG00000133243	BTBD2	-1,082520922
ENSG00000176890	TYMS	-1,077391089
ENSG00000275023	MLLT6	-1,077323619
ENSG00000163812	ZDHHC3	-1,073568414
ENSG00000148400	NOTCH1	-1,073271845
ENSG00000065809	FAM107B	-1,066751796
ENSG00000177663	IL17RA	-1,065435244
ENSG00000141753	IGFBP4	-1,059671274
ENSG00000074370	ATP2A3	-1,053854023
ENSG00000100714	MTHFD1	-1,053516588

ENSG00000196591	HDAC2	-1,051358833
ENSG00000234741	GAS5	-1,046562897
ENSG00000140848	CPNE2	-1,044677105
ENSG00000153094	BCL2L11	-1,042261187
ENSG00000124207	CSE1L	-1,041833129
ENSG00000132780	NASP	-1,038431307
ENSG00000100448	CTSG	-1,03795915
ENSG00000173020	GRK2	-1,035862894
ENSG00000086475	SEPHS1	-1,035847809
ENSG00000147854	UHRF2	-1,024433715
ENSG00000138160	KIF11	-1,020196847
ENSG00000070814	TCOF1	-1,017794795
ENSG00000104738	MCM4	-1,017724914
ENSG00000111144	LTA4H	-1,016639405
ENSG00000131759	RARA	-1,014142921
ENSG00000132383	RPA1	-1,013562877
ENSG00000141510	TP53	-1,011453043
ENSG00000178921	PFAS	-1,011324187
ENSG00000198900	TOP1	1,001869357
ENSG00000136156	ITM2B	1,003674212
ENSG00000143570	SLC39A1	1,01541984
ENSG00000143799	PARP1	1,016430228
ENSG00000171241	SHCBP1	1,026098572
ENSG00000107223	EDF1	1,027728221
ENSG00000167815	PRDX2	1,034036783
ENSG00000132824	SERINC3	1,034900601
ENSG00000090857	PDPR	1,036059756
ENSG00000176853	FAM91A1	1,040025563
ENSG00000068878	PSME4	1,041838662
ENSG00000077232	DNAJC10	1,055162193
ENSG00000006125	AP2B1	1,05517808
ENSG00000112977	DAP	1,055664717
ENSG00000143774	GUK1	1,056523905
ENSG00000110719	TCIRG1	1,057857971
ENSG00000197956	S100A6	1,057894506
ENSG00000143753	DEGS1	1,059334722
ENSG00000204525	HLA-C	1,063697258
ENSG00000105281	SLC1A5	1,067355037
ENSG00000136628	EPRS	1,067973302
ENSG00000130811	EIF3G	1,070253071
ENSG00000166747	AP1G1	1,071129227
ENSG00000118855	MFSD1	1,074632896

ENSG00000204713	TRIM27	1,07500355
ENSG00000175931	UBE2O	1,086353475
ENSG00000174437	ATP2A2	1,086752667
ENSG00000171302	CANT1	1,086978709
ENSG00000213593	TMX2	1,088951146
ENSG00000068028	RASSF1	1,089200801
ENSG00000106261	ZKSCAN1	1,089777704
ENSG00000117410	ATP6V0B	1,089779829
ENSG00000135677	GNS	1,093400653
ENSG00000143761	ARF1	1,099249455
ENSG00000132471	WBP2	1,101319033
ENSG00000108344	PSMD3	1,102239585
ENSG00000117592	PRDX6	1,104607418
ENSG00000130164	LDLR	1,105962684
ENSG00000025796	SEC63	1,106837828
ENSG00000099194	SCD	1,107713286
ENSG00000182718	ANXA2	1,107745265
ENSG00000087086	FTL	1,108145237
ENSG00000176014	TUBB6	1,112017397
ENSG00000092841	MYL6	1,115604997
ENSG00000124198	ARFGEF2	1,116292868
ENSG00000104331	IMPAD1	1,123895983
ENSG00000029364	SLC39A9	1,128717862
ENSG00000111961	SASH1	1,129480228
ENSG00000169429	CXCL8	1,130117293
ENSG00000179632	MAF1	1,131970498
ENSG00000077147	TM9SF3	1,132258103
ENSG00000110917	MLEC	1,137071732
ENSG00000078808	SDF4	1,143188521
ENSG00000001629	ANKIB1	1,147802803
ENSG00000113194	FAF2	1,150007206
ENSG00000101150	TPD52L2	1,155378062
ENSG00000071553	ATP6AP1	1,155660858
ENSG00000114902	SPCS1	1,156687293
ENSG00000077380	DYNC1I2	1,158449351
ENSG00000126432	PRDX5	1,161227493
ENSG00000100979	PLTP	1,16638659
ENSG00000185359	HGS	1,173430382
ENSG00000100031	GGT1	1,185915173
ENSG00000089327	FXYD5	1,187176306
ENSG00000143226	FCGR2A	1,193633592
ENSG00000126934	MAP2K2	1,194514933

ENSG00000120889	TNFRSF10B	1,200107831
ENSG00000176171	BNIP3	1,203576287
ENSG00000001631	KRIT1	1,212814001
ENSG00000100029	PES1	1,218277119
ENSG00000104388	RAB2A	1,218832699
ENSG00000113716	HMGXB3	1,223407899
ENSG00000196396	PTPN1	1,233473738
ENSG00000113282	CLINT1	1,23672813
ENSG00000156256	USP16	1,241738326
ENSG00000183291	SELENOF	1,242641898
ENSG00000142227	EMP3	1,243169309
ENSG00000196199	MPHOSPH8	1,243557518
ENSG00000104549	SQLE	1,246025666
ENSG00000162852	CNST	1,247906195
ENSG00000147526	TACC1	1,250095589
ENSG00000198363	ASPH	1,252763913
ENSG00000140264	SERF2	1,253935836
ENSG00000163191	S100A11	1,255017733
ENSG00000158869	FCER1G	1,256988222
ENSG00000186480	INSIG1	1,258743261
ENSG00000065548	ZC3H15	1,261192381
ENSG00000052841	TTC17	1,261744028
ENSG00000127022	CANX	1,265842449
ENSG00000002549	LAP3	1,267529791
ENSG00000105856	HBP1	1,268034341
ENSG00000136758	YME1L1	1,268202582
ENSG00000115977	AAK1	1,270102628
ENSG00000133193	FAM104A	1,272200843
ENSG00000188677	PARVB	1,274329495
ENSG00000281649	EBLN3P	1,276765238
ENSG00000069956	MAPK6	1,28234259
ENSG00000141582	CBX4	1,282539652
ENSG00000116857	TMEM9	1,288444576
ENSG00000185624	P4HB	1,291497499
ENSG00000154832	CXXC1	1,292444087
ENSG00000091542	ALKBH5	1,302138018
ENSG00000067369	TP53BP1	1,307135453
ENSG00000145725	PPIP5K2	1,30716792
ENSG00000141644	MBD1	1,307280484
ENSG00000148606	POLR3A	1,309467542
ENSG00000196352	CD55	1,309572661
ENSG00000175582	RAB6A	1,319077199

ENSG00000166557	TMED3	1,320799761
ENSG00000131446	MGAT1	1,320899334
ENSG00000008294	SPAG9	1,323401156
ENSG00000176986	SEC24C	1,324563305
ENSG00000101782	RIOK3	1,327652178
ENSG00000123983	ACSL3	1,332535004
ENSG00000100994	PYGB	1,33429673
ENSG00000159199	ATP5G1	1,342733779
ENSG00000168282	MGAT2	1,345800896
ENSG00000167615	LENG8	1,347104435
ENSG00000140105	WARS	1,348991598
ENSG00000105355	PLIN3	1,350297324
ENSG00000112514	CUTA	1,350483311
ENSG00000149792	MRPL49	1,350865149
ENSG00000107959	PITRM1	1,35286788
ENSG00000121931	LRIF1	1,352876168
ENSG00000160991	ORAI2	1,354199574
ENSG00000089063	TMEM230	1,354940554
ENSG00000084652	TXLNA	1,365843493
ENSG00000173113	TRMT112	1,367436954
ENSG00000244038	DDOST	1,37112036
ENSG00000125651	GTF2F1	1,374100852
ENSG00000161011	SQSTM1	1,378537082
ENSG00000172115	CYCS	1,381409088
ENSG00000144791	LIMD1	1,381646558
ENSG00000170348	TMED10	1,387371137
ENSG00000181192	DHTKD1	1,38841464
ENSG00000130559	CAMSAP1	1,393450074
ENSG00000130779	CLIP1	1,393717354
ENSG00000104969	SGTA	1,394603425
ENSG00000138674	SEC31A	1,397042144
ENSG00000102226	USP11	1,397970711
ENSG00000168397	ATG4B	1,398654673
ENSG00000110315	RNF141	1,399652667
ENSG00000088832	FKBP1A	1,403031707
ENSG00000049449	RCN1	1,404189694
ENSG00000078747	ITCH	1,404211154
ENSG00000106153	CHCHD2	1,406344534
ENSG00000198301	SDAD1	1,409740849
ENSG00000187051	RPS19BP1	1,410864662
ENSG00000061987	MON2	1,410947872
ENSG00000173545	ZNF622	1,411267594

ENSG00000143621	ILF2	1,411622008
ENSG00000100097	LGALS1	1,414114543
ENSG00000112511	PHF1	1,419361667
ENSG00000141756	FKBP10	1,419983447
ENSG00000107651	SEC23IP	1,422467172
ENSG00000169223	LMAN2	1,424527661
ENSG00000161204	ABCF3	1,425779914
ENSG00000159167	STC1	1,430009934
ENSG00000198466	ZNF587	1,437784802
ENSG00000102893	PHKB	1,442476471
ENSG00000065000	AP3D1	1,446300012
ENSG00000160789	LMNA	1,447279944
ENSG00000135249	RINT1	1,448123406
ENSG00000142669	SH3BGRL3	1,450515289
ENSG00000157637	SLC38A10	1,455151529
ENSG00000143183	TMCO1	1,455729788
ENSG00000213719	CLIC1	1,458184161
ENSG00000106683	LIMK1	1,459251546
ENSG00000168268	NT5DC2	1,467701557
ENSG00000071537	SEL1L	1,468794395
ENSG00000072042	RDH11	1,471815435
ENSG00000137996	RTCA	1,471967554
ENSG00000106348	IMPDH1	1,474184592
ENSG00000172432	GTPBP2	1,476354583
ENSG00000167842	MIS12	1,476715181
ENSG00000149577	SIDT2	1,481081385
ENSG00000164211	STARD4	1,483838837
ENSG00000140688	C16orf58	1,485774048
ENSG00000204356	NELFE	1,48639306
ENSG00000010270	STARD3NL	1,494913933
ENSG00000145391	SETD7	1,495011725
ENSG00000147162	OGT	1,495907365
ENSG00000086062	B4GALT1	1,500686613
ENSG00000011422	PLAUR	1,501892376
ENSG00000100938	GMPR2	1,504411471
ENSG00000277443	MARCKS	1,504823656
ENSG00000168610	STAT3	1,505024222
ENSG00000077235	GTF3C1	1,505768702
ENSG00000276600	RAB7B	1,506252453
ENSG00000088826	SMOX	1,507646188
ENSG00000187688	TRPV2	1,509867357
ENSG00000133318	RTN3	1,511698524

ENSG00000036054	TBC1D23	1,514558101
ENSG00000125734	GPR108	1,517669762
ENSG00000099331	MYO9B	1,518424017
ENSG00000174780	SRP72	1,522799304
ENSG00000147649	MTDH	1,524516666
ENSG00000008952	SEC62	1,525568719
ENSG00000132842	AP3B1	1,525744661
ENSG00000014123	UFL1	1,52699038
ENSG00000196535	MYO18A	1,529850522
ENSG00000160209	PDXK	1,531564614
ENSG00000182220	ATP6AP2	1,537228235
ENSG00000175137	SH3BP5L	1,539732773
ENSG00000118363	SPCS2	1,542039231
ENSG00000104805	NUCB1	1,542554809
ENSG00000169976	SF3B5	1,544700386
ENSG00000182827	ACBD3	1,54604191
ENSG00000129083	COPB1	1,547202782
ENSG00000129317	PUS7L	1,547764766
ENSG00000134851	TMEM165	1,548768568
ENSG00000143222	UFC1	1,553643945
ENSG00000111540	RAB5B	1,56115532
ENSG00000133169	BEX1	1,562227347
ENSG00000182670	TTC3	1,565412068
ENSG00000197217	ENTPD4	1,567254437
ENSG00000100412	ACO2	1,570810407
ENSG00000167460	TPM4	1,573616855
ENSG00000138069	RAB1A	1,578995911
ENSG00000060558	GNA15	1,57970625
ENSG00000197930	ERO1A	1,583330192
ENSG00000106638	TBL2	1,584145137
ENSG00000182325	FBXL6	1,586751815
ENSG00000159921	GNE	1,587437051
ENSG00000100991	TRPC4AP	1,588208653
ENSG00000052802	MSMO1	1,592086959
ENSG00000109572	CLCN3	1,594295078
ENSG00000198417	MT1F	1,603058274
ENSG00000168404	MLKL	1,603743443
ENSG00000133835	HSD17B4	1,603788439
ENSG00000129484	PARP2	1,610843515
ENSG00000183486	MX2	1,611803237
ENSG00000197746	PSAP	1,612673916
ENSG00000179218	CALR	1,613103677

ENSG00000087074	PPP1R15A	1,614035128
ENSG00000118705	RPN2	1,61475766
ENSG00000082996	RNF13	1,616321431
ENSG00000035928	RFC1	1,623453237
ENSG00000257093	KIAA1147	1,626414216
ENSG00000198862	LTN1	1,630071432
ENSG00000173852	DPY19L1	1,634052166
ENSG00000167881	SRP68	1,636312768
ENSG00000145354	CISD2	1,636824292
ENSG00000162910	MRPL55	1,639478713
ENSG00000197157	SND1	1,645813451
ENSG00000094804	CDC6	1,647713165
ENSG00000079332	SAR1A	1,649729523
ENSG00000117500	TMED5	1,652058194
ENSG00000105438	KDELR1	1,653113792
ENSG00000051108	HERPUD1	1,655445528
ENSG00000143515	ATP8B2	1,656975589
ENSG00000162734	PEA15	1,657625798
ENSG00000143870	PDIA6	1,659288397
ENSG00000128059	PPAT	1,659793418
ENSG00000142875	PRKACB	1,661176822
ENSG00000179562	GCC1	1,663332949
ENSG00000144674	GOLGA4	1,666745309
ENSG00000145740	SLC30A5	1,678435835
ENSG00000184014	DENND5A	1,678634704
ENSG00000070495	JMJD6	1,682227427
ENSG00000148248	SURF4	1,683220995
ENSG00000101384	JAG1	1,686688456
ENSG00000169826	CSGALNACT2	1,689000168
ENSG00000022840	RNF10	1,689039384
ENSG00000096070	BRPF3	1,689572207
ENSG00000177683	THAP5	1,689718472
ENSG00000134970	TMED7	1,691983226
ENSG00000128699	ORMDL1	1,692187517
ENSG00000134375	TIMM17A	1,692417147
ENSG00000044115	CTNNA1	1,693094972
ENSG00000090006	LTBP4	1,694193212
ENSG00000095139	ARCN1	1,695589531
ENSG00000154305	MIA3	1,696180196
ENSG00000117385	P3H1	1,696798443
ENSG00000099814	CEP170B	1,698694828
ENSG00000122882	ECD	1,700522467

ENSG00000121073	SLC35B1	1,701156645
ENSG00000144747	TMF1	1,703513764
ENSG00000136026	CKAP4	1,703845527
ENSG00000112078	KCTD20	1,705283356
ENSG00000130733	YIPF2	1,710381314
ENSG00000081307	UBA5	1,717551746
ENSG00000136813	KIAA0368	1,721901303
ENSG00000119632	IFI27L2	1,722550215
ENSG00000106400	ZNHIT1	1,725414408
ENSG00000115317	HTRA2	1,731230606
ENSG00000171311	EXOSC1	1,731591505
ENSG00000111711	GOLT1B	1,731595248
ENSG00000189067	LITAF	1,732096573
ENSG00000117984	CTSD	1,736263113
ENSG00000144224	UBXN4	1,736639044
ENSG00000104957	CCDC130	1,740716935
ENSG00000126524	SBDS	1,744310281
ENSG00000177951	BET1L	1,744497941
ENSG00000142186	SCYL1	1,74541189
ENSG00000109854	HTATIP2	1,750107745
ENSG00000062716	VMP1	1,757327209
ENSG00000119912	IDE	1,758056045
ENSG00000135046	ANXA1	1,758458081
ENSG00000112972	HMGCS1	1,772437873
ENSG00000136868	SLC31A1	1,781123343
ENSG00000033627	ATP6V0A1	1,784451982
ENSG00000102007	PLP2	1,785119054
ENSG00000213699	SLC35F6	1,78904259
ENSG00000111678	C12orf57	1,789304528
ENSG00000162236	STX5	1,791281987
ENSG00000181704	YIPF6	1,791431366
ENSG00000133398	MED10	1,791634532
ENSG00000139182	CLSTN3	1,799666404
ENSG00000170876	TMEM43	1,800400339
ENSG00000164040	PGRMC2	1,801120351
ENSG00000130589	HELZ2	1,802803008
ENSG00000204438	GPANK1	1,804272313
ENSG00000228474	OST4	1,812249206
ENSG00000154813	DPH3	1,814455126
ENSG00000140650	PMM2	1,814994913
ENSG00000100647	SUSD6	1,815221134
ENSG00000072778	ACADVL	1,817739254

ENSG00000128891	CCDC32	1,820637324
ENSG00000123562	MORF4L2	1,821253964
ENSG00000177888	ZBTB41	1,821684876
ENSG00000151779	NBAS	1,827131298
ENSG00000101194	SLC17A9	1,827382982
ENSG00000109065	NAT9	1,829900561
ENSG00000104412	EMC2	1,83386676
ENSG00000160058	BSDC1	1,839187521
ENSG00000101310	SEC23B	1,844652995
ENSG00000135404	CD63	1,850434249
ENSG00000166794	PPIB	1,856120466
ENSG00000182054	IDH2	1,859289563
ENSG00000104164	BLOC1S6	1,862334741
ENSG00000130703	OSBPL2	1,86634472
ENSG00000167778	SPRYD3	1,86844234
ENSG00000013275	PSMC4	1,868507984
ENSG00000143575	HAX1	1,87098099
ENSG00000059728	MXD1	1,871403935
ENSG00000101350	KIF3B	1,871974644
ENSG00000115275	MOGS	1,873106663
ENSG00000268758	ADGRE4P	1,873918749
ENSG00000122218	COPA	1,877408601
ENSG00000119523	ALG2	1,878799031
ENSG00000198961	PJA2	1,882236005
ENSG00000171109	MFN1	1,88269756
ENSG00000197150	ABCB8	1,891545158
ENSG00000125868	DSTN	1,897874016
ENSG00000083223	ZCCHC6	1,897972682
ENSG00000125459	MSTO1	1,899908955
ENSG00000055147	FAM114A2	1,903784191
ENSG00000070081	NUCB2	1,914692745
ENSG00000125037	EMC3	1,915771306
ENSG00000214655	ZSWIM8	1,920910442
ENSG00000138942	RNF185	1,922722259
ENSG00000167123	CERCAM	1,924141305
ENSG00000105669	COPE	1,92474606
ENSG00000156639	ZFAND3	1,926989003
ENSG00000134910	STT3A	1,928347046
ENSG00000129562	DAD1	1,929423583
ENSG00000068438	FTSJ1	1,935155372
ENSG00000114354	TFG	1,936621307
ENSG00000113621	TXNDC15	1,940520788

ENSG00000168894	RNF181	1,941626885
ENSG00000060762	MPC1	1,948140701
ENSG00000087087	SRRT	1,948196326
ENSG00000125148	MT2A	1,956840602
ENSG00000198162	MAN1A2	1,961944678
ENSG00000112305	SMAP1	1,962223203
ENSG00000140941	MAP1LC3B	1,969596796
ENSG00000059804	SLC2A3	1,974424203
ENSG00000196937	FAM3C	1,974800722
ENSG00000135506	OS9	1,975244311
ENSG00000173486	FKBP2	1,975560468
ENSG00000179119	SPTY2D1	1,975606293
ENSG00000108039	XPNPEP1	1,97682125
ENSG00000023318	ERP44	1,977039123
ENSG00000104635	SLC39A14	1,978944838
ENSG00000108405	P2RX1	1,980785783
ENSG00000110048	OSBP	1,984268863
ENSG00000152484	USP12	1,985529745
ENSG00000033100	CHPF2	1,990690659
ENSG00000113615	SEC24A	1,997987778
ENSG00000204178	TMEM57	2,002301087
ENSG00000102871	TRADD	2,004414893
ENSG00000070061	ELP1	2,004998693
ENSG00000110660	SLC35F2	2,009056703
ENSG00000074842	MYDGF	2,011474133
ENSG00000166130	IKBIP	2,013812501
ENSG00000110344	UBE4A	2,016166867
ENSG00000184432	COPB2	2,017759361
ENSG00000135048	TMEM2	2,022583107
ENSG00000132196	HSD17B7	2,02414091
ENSG00000180229	HERC2P3	2,028690772
ENSG00000125629	INSIG2	2,031661612
ENSG00000086598	TMED2	2,031767574
ENSG00000171928	TVP23B	2,033264879
ENSG00000171631	P2RY6	2,036528406
ENSG00000136986	DERL1	2,037218244
ENSG00000166598	HSP90B1	2,040620375
ENSG00000097033	SH3GLB1	2,041077298
ENSG00000126903	SLC10A3	2,041332114
ENSG00000189114	BLOC1S3	2,042440392
ENSG00000100284	TOM1	2,043135419
ENSG00000174938	SEZ6L2	2,043818832

ENSG00000123395	ATG101	2,050950163
ENSG00000167378	IRGQ	2,061029151
ENSG00000088970	KIZ	2,0652077
ENSG00000138768	USO1	2,068827107
ENSG00000094975	SUCO	2,075040655
ENSG00000163818	LZTFL1	2,07506607
ENSG00000066455	GOLGA5	2,075785621
ENSG00000115461	IGFBP5	2,077747532
ENSG00000198722	UNC13B	2,078086187
ENSG00000115677	HDLBP	2,081097416
ENSG00000139637	C12orf10	2,082444682
ENSG00000050405	LIMA1	2,083225809
ENSG00000107862	GBF1	2,084683405
ENSG00000174851	YIF1A	2,093974908
ENSG00000108433	GOSR2	2,095126686
ENSG00000105321	CCDC9	2,105001035
ENSG00000118596	SLC16A7	2,1069709
ENSG00000105829	BET1	2,11056995
ENSG00000265808	SEC22B	2,112207007
ENSG00000072415	MPP5	2,115199516
ENSG00000168615	ADAM9	2,119025246
ENSG00000124762	CDKN1A	2,119818348
ENSG00000114251	WNT5A	2,124930316
ENSG00000100116	GCAT	2,127085569
ENSG00000120805	ARL1	2,132002338
ENSG00000197747	S100A10	2,134120737
ENSG00000172716	SLFN11	2,136170239
ENSG00000165280	VCP	2,142609238
ENSG00000171161	ZNF672	2,142613517
ENSG00000103489	XYLT1	2,15352892
ENSG00000168495	POLR3D	2,154621652
ENSG00000173221	GLRX	2,15787941
ENSG00000034510	TMSB10	2,159735073
ENSG00000120708	TGFBI	2,160064174
ENSG00000160691	SHC1	2,168376811
ENSG00000117475	BLZF1	2,16843327
ENSG00000173875	ZNF791	2,171831764
ENSG00000198856	OSTC	2,172025492
ENSG00000162909	CAPN2	2,173726147
ENSG00000096654	ZNF184	2,17694498
ENSG00000101199	ARFGAP1	2,177854635
ENSG00000054523	KIF1B	2,178598268

ENSG00000166471	TMEM41B	2,179256758
ENSG00000091592	NLRP1	2,180552131
ENSG00000132254	ARFIP2	2,191309106
ENSG00000196544	BORCS6	2,194422231
ENSG00000151012	SLC7A11	2,196032703
ENSG00000117143	UAP1	2,199290629
ENSG00000177606	JUN	2,202283817
ENSG00000120519	SLC10A7	2,202466577
ENSG00000181789	COPG1	2,209890414
ENSG00000140854	KATNB1	2,210348861
ENSG00000135631	RAB11FIP5	2,21794128
ENSG00000153989	NUS1	2,22210685
ENSG00000171867	PRNP	2,223528961
ENSG00000078246	TULP3	2,231920827
ENSG00000155850	SLC26A2	2,232590561
ENSG00000075420	FNDC3B	2,238209482
ENSG00000133872	SARAF	2,239448295
ENSG00000156958	GALK2	2,241822611
ENSG00000167004	PDIA3	2,243355851
ENSG00000159023	EPB41	2,246384364
ENSG00000114395	CYB561D2	2,26116821
ENSG00000090615	GOLGA3	2,262264837
ENSG00000130827	PLXNA3	2,275229211
ENSG00000116717	GADD45A	2,276537084
ENSG00000158467	AHCYL2	2,281681675
ENSG00000101017	CD40	2,284647113
ENSG00000138073	PREB	2,294674815
ENSG00000052749	RRP12	2,295858286
ENSG00000123131	PRDX4	2,300822113
ENSG00000100554	ATP6V1D	2,303641814
ENSG00000163479	SSR2	2,307085007
ENSG00000108829	LRRC59	2,314964922
ENSG00000076043	REXO2	2,316471362
ENSG00000164244	PRRC1	2,319908057
ENSG00000120742	SERP1	2,322745254
ENSG00000167645	YIF1B	2,324626061
ENSG00000026950	BTN3A1	2,326536577
ENSG00000078177	N4BP2	2,329163847
ENSG00000141696	P3H4	2,332126051
ENSG00000157020	SEC13	2,333809997
ENSG00000172057	ORMDL3	2,333857028
ENSG00000167110	GOLGA2	2,344500188

ENSG00000100934	SEC23A	2,345781863
ENSG00000173230	GOLGB1	2,347999815
ENSG00000259803	SLC22A31	2,348288193
ENSG00000106636	YKT6	2,354548003
ENSG00000167977	KCTD5	2,357560722
ENSG00000086619	ERO1B	2,359843945
ENSG00000104825	NFKBIB	2,362738719
ENSG00000170946	DNAJC24	2,370616632
ENSG00000180879	SSR4	2,375004523
ENSG00000125844	RRBP1	2,375218262
ENSG00000168701	TMEM208	2,380279878
ENSG00000075399	VPS9D1	2,387949653
ENSG00000132432	SEC61G	2,392759228
ENSG00000154640	BTG3	2,394069973
ENSG00000179454	KLHL28	2,399088533
ENSG00000140598	EFL1	2,407548158
ENSG00000119777	TMEM214	2,412713933
ENSG00000120725	SIL1	2,412739182
ENSG00000184840	TMED9	2,422572055
ENSG00000140939	NOL3	2,429184023
ENSG00000114439	BBX	2,431379433
ENSG00000143751	SDE2	2,435698816
ENSG00000110697	PITPNM1	2,435982966
ENSG00000132256	TRIM5	2,445112897
ENSG00000072849	DERL2	2,451856602
ENSG00000004866	ST7	2,454473503
ENSG00000197620	CXorf40A	2,460693362
ENSG00000119729	RHOQ	2,474947791
ENSG00000176018	LYSMD3	2,47878303
ENSG00000155304	HSPA13	2,48217452
ENSG00000159720	ATP6V0D1	2,487555492
ENSG00000005700	IBTK	2,489090237
ENSG00000166579	NDEL1	2,492315773
ENSG00000130202	NECTIN2	2,495443107
ENSG00000136643	RPS6KC1	2,499527121
ENSG00000182934	SRPRA	2,503958487
ENSG00000102158	MAGT1	2,507992119
ENSG00000242247	ARFGAP3	2,52483497
ENSG00000183718	TRIM52	2,529838565
ENSG00000100612	DHRS7	2,539876165
ENSG00000163902	RPN1	2,541327264
ENSG00000074695	LMAN1	2,553396069

ENSG00000112473	SLC39A7	2,556979406
ENSG00000119812	FAM98A	2,558584215
ENSG00000182197	EXT1	2,570477123
ENSG00000128335	APOL2	2,572494511
ENSG00000204386	NEU1	2,578741255
ENSG00000017483	SLC38A5	2,588833854
ENSG00000100883	SRP54	2,598669974
ENSG00000187792	ZNF70	2,605749458
ENSG00000184500	PROS1	2,622806941
ENSG00000103966	EHD4	2,630483659
ENSG00000120256	LRP11	2,631031455
ENSG00000184164	CRELD2	2,634150845
ENSG00000127526	SLC35E1	2,641600956
ENSG00000068971	PPP2R5B	2,65179931
ENSG00000141504	SAT2	2,656907381
ENSG00000135953	MFSD9	2,658630603
ENSG00000124357	NAGK	2,664248942
ENSG00000044574	HSPA5	2,669283686
ENSG00000165169	DYNLT3	2,678649349
ENSG00000169359	SLC33A1	2,68124469
ENSG00000180398	MCFD2	2,686912446
ENSG00000090520	DNAJB11	2,692466948
ENSG00000196663	TECPR2	2,695854267
ENSG00000106803	SEC61B	2,711155864
ENSG00000185803	SLC52A2	2,717141067
ENSG00000143457	GOLPH3L	2,730006325
ENSG00000011478	QPCTL	2,730301003
ENSG00000068697	LAPTM4A	2,733135188
ENSG00000168685	IL7R	2,734213775
ENSG00000173706	HEG1	2,739251313
ENSG00000145817	YIPF5	2,784279012
ENSG00000058262	SEC61A1	2,791333452
ENSG00000128228	SDF2L1	2,791970977
ENSG00000124783	SSR1	2,797518875
ENSG00000144867	SRPRB	2,824062137
ENSG00000163644	PPM1K	2,825363881
ENSG00000135241	PNPLA8	2,83212248
ENSG00000065485	PDIA5	2,834405273
ENSG00000176845	METRNL	2,856728566
ENSG00000134324	LPIN1	2,858851305
ENSG00000167797	CDK2AP2	2,864650471
ENSG00000196220	SRGAP3	2,868835692

ENSG00000130066	SAT1	2,869228023
ENSG00000067167	TRAM1	2,872910403
ENSG00000115963	RND3	2,879449261
ENSG00000173110	HSPA6	2,882596124
ENSG00000102531	FNDC3A	2,891234833
ENSG00000147872	PLIN2	2,891307683
ENSG00000162298	SYVN1	2,892331572
ENSG00000254858	MPV17L2	2,893353673
ENSG00000197903	HIST1H2BK	2,893927394
ENSG00000068903	SIRT2	2,894638033
ENSG00000165948	IFI27L1	2,898275295
ENSG00000198380	GFPT1	2,922062723
ENSG00000162695	SLC30A7	2,925283841
ENSG00000168374	ARF4	2,927168416
ENSG00000105835	NAMPT	2,927214265
ENSG00000083444	PLOD1	2,929433294
ENSG00000095380	NANS	2,932439291
ENSG00000115806	GORASP2	2,951121941
ENSG00000072952	MRVI1	2,967089545
ENSG00000179832	MROH1	2,979977012
ENSG00000197712	FAM114A1	2,984474687
ENSG00000178104	PDE4DIP	2,987457549
ENSG00000073350	LLGL2	2,989753238
ENSG00000182022	CHST15	2,994650279
ENSG00000151914	DST	3,002912451
ENSG00000144591	GMPPA	3,010810203
ENSG00000176142	TMEM39A	3,015779398
ENSG00000136240	KDELR2	3,018934491
ENSG00000120686	UFM1	3,020146206
ENSG00000145050	MANF	3,026395194
ENSG00000101294	HM13	3,036299393
ENSG00000138448	ITGAV	3,045617842
ENSG00000136010	ALDH1L2	3,052567697
ENSG00000158373	HIST1H2BD	3,080582243
ENSG00000170955	CAVIN3	3,082319617
ENSG00000151929	BAG3	3,093243175
ENSG00000147400	CETN2	3,095878173
ENSG00000145555	MYO10	3,107213054
ENSG00000169241	SLC50A1	3,113255064
ENSG00000088298	EDEM2	3,114119577
ENSG00000221926	TRIM16	3,148960515
ENSG00000150961	SEC24D	3,161996685

ENSG00000167680	SEMA6B	3,16290749
ENSG00000172071	EIF2AK3	3,180399598
ENSG00000128595	CALU	3,19486019
ENSG00000135924	DNAJB2	3,204130873
ENSG00000197632	SERPINB2	3,210947482
ENSG00000089127	OAS1	3,2215146
ENSG00000102401	ARMCX3	3,227241195
ENSG00000187266	EPOR	3,234718934
ENSG00000149428	HYOU1	3,235917308
ENSG00000173540	GMPPB	3,240842716
ENSG00000151135	TMEM263	3,25423764
ENSG00000155660	PDIA4	3,263632891
ENSG00000173559	NABP1	3,265422021
ENSG00000136770	DNAJC1	3,275205715
ENSG00000114850	SSR3	3,300890203
ENSG00000087903	RFX2	3,303410183
ENSG00000131871	SELENOS	3,303825109
ENSG00000178381	ZFAND2A	3,334881643
ENSG00000166562	SEC11C	3,35695325
ENSG00000163814	CDCP1	3,371319334
ENSG00000139112	GABARAPL1	3,373300192
ENSG00000125388	GRK4	3,378787496
ENSG00000117682	DHDDS	3,402068001
ENSG00000164674	SYTL3	3,424748929
ENSG00000068912	ERLEC1	3,425685344
ENSG00000170448	NFXL1	3,432479639
ENSG00000215154	AC141586.1	3,439782289
ENSG00000107175	CREB3	3,456263712
ENSG00000144655	CSRNP1	3,477928761
ENSG00000013375	PGM3	3,478974518
ENSG00000102580	DNAJC3	3,479158013
ENSG00000214530	STARD10	3,484154094
ENSG00000160219	GAB3	3,499452558
ENSG00000121940	CLCC1	3,500255593
ENSG00000150403	TMCO3	3,504298872
ENSG00000143067	ZNF697	3,507439084
ENSG00000012822	CALCOCO1	3,526563649
ENSG00000070540	WIPI1	3,530976931
ENSG00000181982	CCDC149	3,560290278
ENSG00000079385	CEACAM1	3,560891787
ENSG00000134285	FKBP11	3,600895172
ENSG00000101298	SNPH	3,604495125

ENSG00000010278	CD9	3,612656705
ENSG00000181035	SLC25A42	3,663624803
ENSG00000072682	P4HA2	3,694154498
ENSG00000113811	SELENOK	3,694411802
ENSG00000185015	CA13	3,713753832
ENSG00000164111	ANXA5	3,81804291
ENSG00000163683	SMIM14	3,879105511
ENSG00000062524	LTK	3,893328829
ENSG00000142552	RCN3	3,942502018
ENSG00000139793	MBNL2	3,955258654
ENSG00000139116	KIF21A	3,959049579
ENSG00000074855	ANO8	3,978258588
ENSG00000162520	SYNC	3,986162715
ENSG00000136840	ST6GALNAC4	3,993033067
ENSG00000184205	TSPYL2	3,997176385
ENSG00000085514	PILRA	4,007015592
ENSG00000105464	GRIN2D	4,014209009
ENSG00000138678	GPAT3	4,016827339
ENSG00000123989	CHPF	4,033193118
ENSG00000156711	MAPK13	4,048852515
ENSG00000085733	CTTN	4,119666047
ENSG00000135709	KIAA0513	4,131531597
ENSG00000197496	SLC2A10	4,156258075
ENSG00000148926	ADM	4,16790203
ENSG00000173451	THAP2	4,195097166
ENSG00000182118	FAM89A	4,199856974
ENSG00000106733	NMRK1	4,222824452
ENSG00000140961	OSGIN1	4,242423296
ENSG00000163249	CCNYL1	4,246184666
ENSG00000181649	PHLDA2	4,246345908
ENSG00000170379	TCAF2	4,257675945
ENSG00000164023	SGMS2	4,282196355
ENSG00000010310	GIPR	4,289468217
ENSG00000198682	PAPSS2	4,303526128
ENSG00000180061	TMEM150B	4,326091558
ENSG00000164949	GEM	4,338374018
ENSG00000184988	TMEM106A	4,355285343
ENSG00000099337	KCNK6	4,372553349
ENSG00000003147	ICA1	4,398342719
ENSG00000166816	LDHD	4,461066986
ENSG00000147535	PLPP5	4,551886867
ENSG00000105404	RABAC1	4,579212795

ENSG00000120049	KCNIP2	4,598338549
ENSG00000106080	FKBP14	4,624974291
ENSG00000259343	TMC3-AS1	4,638185842
ENSG00000153234	NR4A2	4,696147446
ENSG00000173531	MST1	4,735338117
ENSG00000146426	TIAM2	4,753819043
ENSG00000099954	CECR2	4,78584955
ENSG00000198018	ENTPD7	4,852383193
ENSG00000174837	ADGRE1	4,856186476
ENSG00000147174	GCNA	4,866116
ENSG00000162069	BICDL2	4,958144526
ENSG00000198753	PLXNB3	4,985651061
ENSG00000167772	ANGPTL4	4,998784414
ENSG00000113083	LOX	5,025356672
ENSG00000099958	DERL3	5,035299076
ENSG00000181458	TMEM45A	5,051353234
ENSG00000165272	AQP3	5,094176592
ENSG00000128165	ADM2	5,347219899
ENSG00000123342	MMP19	5,364030845
ENSG00000186594	MIR22HG	5,407187766
ENSG00000172183	ISG20	5,417972017
ENSG00000205710	C17orf107	5,444282765
ENSG00000214274	ANG	5,475872431
ENSG00000134531	EMP1	5,498250079
ENSG00000185339	TCN2	5,537513088
ENSG00000137941	TTLL7	5,543299736
ENSG00000198855	FICD	5,623524892
ENSG00000091986	CCDC80	5,664805461
ENSG00000131459	GFPT2	5,756247196
ENSG00000128590	DNAJB9	5,798285493
ENSG00000236453	AC003092.1	5,910464553
ENSG00000100219	XBP1	5,91051101
ENSG00000103742	IGDCC4	5,970794443
ENSG00000233452	STXBP5-AS1	5,970801812
ENSG00000164692	COL1A2	6,014389803
ENSG00000248323	LUCAT1	6,028002143
ENSG00000100196	KDELR3	6,061542768
ENSG00000104313	EYA1	6,088779684
ENSG00000109501	WFS1	6,25157174
ENSG00000131015	ULBP2	6,450941811
ENSG00000112379	ARFGEF3	6,479012689
ENSG00000080493	SLC4A4	6,495914021

ENSG00000198832	SELENOM	6,664339689
ENSG00000272405	AL365181.3	6,702190812
ENSG00000215045	GRID2IP	6,761219265
ENSG00000167077	MEI1	6,858287505
ENSG00000153531	ADPRHL1	6,862848396
ENSG00000081803	CADPS2	7,00001674
ENSG00000257605	AC073611.1	7,185064138
ENSG00000108176	DNAJC12	7,250012026
ENSG00000112902	SEMA5A	7,403216889
ENSG00000187800	PEAR1	7,413412264
ENSG00000136052	SLC41A2	7,472728969
ENSG00000130294	KIF1A	7,508848707
ENSG00000090104	RGS1	7,51938095
ENSG00000132938	MTUS2	7,799575397
ENSG00000112299	VNN1	7,837839149
ENSG00000189233	NUGGC	7,920275094
ENSG00000117472	TSPAN1	7,937826322
ENSG00000018625	ATP1A2	7,958128843
ENSG00000154589	LY96	8,30945254
ENSG00000214050	FBXO16	8,375873924
ENSG00000109846	CRYAB	8,418687896
ENSG00000180535	BHLHA15	8,4650519
ENSG00000162551	ALPL	8,582906354
ENSG00000151240	DIP2C	8,598836903
ENSG00000171346	KRT15	8,693582209
ENSG00000182253	SYNM	9,280384122
ENSG00000137573	SULF1	9,546317951
ENSG00000115008	IL1A	9,960377001
ENSG00000211891	IGHE	10,55443074

***Supplementary table 4*: XBP1s direct targets identified by cross-referencing RNA sequencing and ChIP sequencing data**

List of the 458 genes that are directly regulated more than 2-fold upon XBP1s expression in OCI-AML3 XBP1s cells compared to TETON control cells after treatment with 10ng/mL doxycycline for 48H (Fold changes are expressed in log2).

Ensembl ID	Genes Symbol	Fold Change (log2)
ENSG00000115008	IL1A	9,960377001
ENSG00000214050	FBXO16	8,375873924
ENSG00000189233	NUGGC	7,920275094
ENSG00000112299	VNN1	7,837839149
ENSG00000136052	SLC41A2	7,472728969
None	AC073611.1	7,185064138
ENSG00000112379	ARFGEF3	6,479012689
ENSG00000100196	KDELR3	6,061542768
ENSG00000248323	LUCAT1	6,028002143
ENSG00000100219	XBP1	5,91051101
ENSG00000128590	DNAJB9	5,798285493
ENSG00000198855	FICD	5,623524892
ENSG00000134531	EMP1	5,498250079
ENSG00000214274	ANG	5,475872431
ENSG00000205710	C17orf107	5,444282765
ENSG00000186594	MIR22HG	5,407187766
ENSG00000165272	AQP3	5,094176592
ENSG00000181458	TMEM45A	5,051353234
ENSG00000099958	DERL3	5,035299076
ENSG00000113083	LOX	5,025356672
ENSG00000174837	ADGRE1	4,856186476
ENSG00000173531	MST1	4,735338117
ENSG00000153234	NR4A2	4,696147446
ENSG00000259343	TMC3-AS1	4,638185842
ENSG00000106080	FKBP14	4,624974291
ENSG00000105404	RABAC1	4,579212795
ENSG00000147535	PLPP5	4,551886867
ENSG00000099337	KCNK6	4,372553349
ENSG00000184988	TMEM106A	4,355285343
ENSG00000010310	GIPR	4,289468217
ENSG00000164023	SGMS2	4,282196355
ENSG00000163249	CCNYL1	4,246184666
ENSG00000173451	THAP2	4,195097166
ENSG00000156711	MAPK13	4,048852515
ENSG00000123989	CHPF	4,033193118
ENSG00000184205	TSPYL2	3,997176385
ENSG00000136840	ST6GALNAC4	3,993033067
ENSG00000139793	MBNL2	3,955258654
ENSG00000142552	RCN3	3,942502018
ENSG00000062524	LTK	3,893328829
ENSG00000163683	SMIM14	3,879105511

ENSG00000164111	ANXA5	3,81804291
ENSG00000113811	SELENOK	3,694411802
ENSG00000181035	SLC25A42	3,663624803
ENSG00000181982	CCDC149	3,560290278
ENSG00000070540	WIPI1	3,530976931
ENSG00000012822	CALCOCO1	3,526563649
ENSG00000121940	CLCC1	3,500255593
ENSG00000214530	STARD10	3,484154094
ENSG00000102580	DNAJC3	3,479158013
ENSG00000013375	PGM3	3,478974518
ENSG00000107175	CREB3	3,456263712
None	AC141586.1	3,439782289
ENSG00000170448	NFXL1	3,432479639
ENSG00000068912	ERLEC1	3,425685344
ENSG00000117682	DHDDS	3,402068001
ENSG00000139112	GABARAPL1	3,373300192
ENSG00000178381	ZFAND2A	3,334881643
ENSG00000131871	SELENOS	3,303825109
ENSG00000114850	SSR3	3,300890203
ENSG00000173559	NABP1	3,265422021
ENSG00000155660	PDIA4	3,263632891
ENSG00000151135	TMEM263	3,25423764
ENSG00000173540	GMPPB	3,240842716
ENSG00000149428	HYOU1	3,235917308
ENSG00000135924	DNAJB2	3,204130873
ENSG00000128595	CALU	3,19486019
ENSG00000172071	EIF2AK3	3,180399598
ENSG00000088298	EDEM2	3,114119577
ENSG00000169241	SLC50A1	3,113255064
ENSG00000151929	BAG3	3,093243175
ENSG00000136010	ALDH1L2	3,052567697
ENSG00000101294	HM13	3,036299393
ENSG00000145050	MANF	3,026395194
ENSG00000120686	UFM1	3,020146206
ENSG00000136240	KDELR2	3,018934491
ENSG00000144591	GMPPA	3,010810203
ENSG00000197712	FAM114A1	2,984474687
ENSG00000115806	GORASP2	2,951121941
ENSG00000095380	NANS	2,932439291
ENSG00000083444	PLOD1	2,929433294
ENSG00000168374	ARF4	2,927168416
ENSG00000162695	SLC30A7	2,925283841

ENSG00000198380	GFPT1	2,922062723
ENSG00000068903	SIRT2	2,894638033
ENSG00000197903	HIST1H2BK	2,893927394
ENSG00000254858	MPV17L2	2,893353673
ENSG00000162298	SYVN1	2,892331572
ENSG00000173110	HSPA6	2,882596124
ENSG00000067167	TRAM1	2,872910403
ENSG00000196220	SRGAP3	2,868835692
ENSG00000167797	CDK2AP2	2,864650471
ENSG00000065485	PDIA5	2,834405273
ENSG00000135241	PNPLA8	2,83212248
ENSG00000163644	PPM1K	2,825363881
ENSG00000128228	SDF2L1	2,791970977
ENSG00000145817	YIPF5	2,784279012
ENSG00000168685	IL7R	2,734213775
ENSG00000068697	LAPTM4A	2,733135188
ENSG00000011478	QPCTL	2,730301003
ENSG00000143457	GOLPH3L	2,730006325
ENSG00000106803	SEC61B	2,711155864
ENSG00000169359	SLC33A1	2,68124469
ENSG00000044574	HSPA5	2,669283686
ENSG00000135953	MFSD9	2,658630603
ENSG00000127526	SLC35E1	2,641600956
ENSG00000103966	EHD4	2,630483659
ENSG00000184500	PROS1	2,622806941
ENSG00000187792	ZNF70	2,605749458
ENSG00000119812	FAM98A	2,558584215
ENSG00000074695	LMAN1	2,553396069
ENSG00000163902	RPN1	2,541327264
ENSG00000183718	TRIM52	2,529838565
ENSG00000102158	MAGT1	2,507992119
ENSG00000182934	SRPRA	2,503958487
ENSG00000136643	RPS6KC1	2,499527121
ENSG00000005700	IBTK	2,489090237
ENSG00000159720	ATP6V0D1	2,487555492
ENSG00000155304	HSPA13	2,48217452
ENSG00000176018	LYSMD3	2,47878303
ENSG00000119729	RHOQ	2,474947791
None	CXorf40A	2,460693362
ENSG00000072849	DERL2	2,451856602
ENSG00000110697	PITPNM1	2,435982966
ENSG00000143751	SDE2	2,435698816

ENSG00000140939	NOL3	2,429184023
ENSG00000184840	TMED9	2,422572055
ENSG00000119777	TMEM214	2,412713933
ENSG00000140598	EFL1	2,407548158
ENSG00000132432	SEC61G	2,392759228
ENSG00000170946	DNAJC24	2,370616632
ENSG00000104825	NFKBIB	2,362738719
ENSG00000086619	ERO1B	2,359843945
ENSG00000106636	YKT6	2,354548003
ENSG00000173230	GOLGB1	2,347999815
ENSG00000167110	GOLGA2	2,344500188
ENSG00000157020	SEC13	2,333809997
ENSG00000141696	P3H4	2,332126051
ENSG00000164244	PRRC1	2,319908057
ENSG00000108829	LRRC59	2,314964922
ENSG00000163479	SSR2	2,307085007
ENSG00000138073	PREB	2,294674815
ENSG00000158467	AHCYL2	2,281681675
ENSG00000114395	CYB561D2	2,26116821
ENSG00000167004	PDIA3	2,243355851
ENSG00000133872	SARAF	2,239448295
ENSG00000075420	FNDC3B	2,238209482
ENSG00000155850	SLC26A2	2,232590561
ENSG00000171867	PRNP	2,223528961
ENSG00000153989	NUS1	2,22210685
ENSG00000181789	COPG1	2,209890414
ENSG00000120519	SLC10A7	2,202466577
ENSG00000117143	UAP1	2,199290629
ENSG00000151012	SLC7A11	2,196032703
ENSG00000132254	ARFIP2	2,191309106
ENSG00000166471	TMEM41B	2,179256758
ENSG00000101199	ARFGAP1	2,177854635
ENSG00000096654	ZNF184	2,17694498
ENSG00000198856	OSTC	2,172025492
ENSG00000173875	ZNF791	2,171831764
ENSG00000117475	BLZF1	2,16843327
ENSG00000160691	SHC1	2,168376811
ENSG00000173221	GLRX	2,15787941
ENSG00000168495	POLR3D	2,154621652
ENSG00000103489	XYLT1	2,15352892
ENSG00000165280	VCP	2,142609238
ENSG00000120805	ARL1	2,132002338

ENSG00000100116	GCAT	2,127085569
ENSG00000265808	SEC22B	2,112207007
ENSG00000105829	BET1	2,11056995
ENSG00000118596	SLC16A7	2,1069709
ENSG00000108433	GOSR2	2,095126686
ENSG00000174851	YIF1A	2,093974908
ENSG00000107862	GBF1	2,084683405
ENSG00000050405	LIMA1	2,083225809
None	C12orf10	2,082444682
ENSG00000198722	UNC13B	2,078086187
ENSG00000066455	GOLGA5	2,075785621
ENSG00000094975	SUCO	2,075040655
ENSG00000138768	USO1	2,068827107
ENSG00000088970	KIZ	2,0652077
ENSG00000167378	IRGQ	2,061029151
ENSG00000123395	ATG101	2,050950163
ENSG00000174938	SEZ6L2	2,043818832
ENSG00000100284	TOM1	2,043135419
ENSG00000189114	BLOC1S3	2,042440392
ENSG00000166598	HSP90B1	2,040620375
ENSG00000136986	DERL1	2,037218244
ENSG00000171928	TVP23B	2,033264879
ENSG00000086598	TMED2	2,031767574
ENSG00000125629	INSIG2	2,031661612
ENSG00000290376	HERC2P3	2,028690772
ENSG00000132196	HSD17B7	2,02414091
ENSG00000184432	COPB2	2,017759361
ENSG00000110344	UBE4A	2,016166867
ENSG00000166130	IKBIP	2,013812501
ENSG00000074842	MYDGF	2,011474133
ENSG00000070061	ELP1	2,004998693
ENSG00000102871	TRADD	2,004414893
ENSG00000204178	MACO1	2,002301087
ENSG00000113615	SEC24A	1,997987778
ENSG00000110048	OSBP	1,984268863
ENSG00000023318	ERP44	1,977039123
ENSG00000179119	SPTY2D1	1,975606293
ENSG00000173486	FKBP2	1,975560468
ENSG00000135506	OS9	1,975244311
ENSG00000196937	FAM3C	1,974800722
ENSG00000112305	SMAP1	1,962223203
ENSG00000168894	RNF181	1,941626885

ENSG00000114354	TFG	1,936621307
ENSG00000068438	FTSJ1	1,935155372
ENSG00000129562	DAD1	1,929423583
ENSG00000134910	STT3A	1,928347046
ENSG00000105669	COPE	1,92474606
ENSG00000055147	FAM114A2	1,903784191
ENSG00000125459	MSTO1	1,899908955
ENSG00000197150	ABCB8	1,891545158
ENSG00000171109	MFN1	1,88269756
ENSG00000198961	PJA2	1,882236005
ENSG00000119523	ALG2	1,878799031
ENSG00000122218	COPA	1,877408601
ENSG00000268758	ADGRE4P	1,873918749
ENSG00000115275	MOGS	1,873106663
ENSG00000143575	HAX1	1,87098099
ENSG00000013275	PSMC4	1,868507984
ENSG00000167778	SPRYD3	1,86844234
ENSG00000104164	BLOC1S6	1,862334741
ENSG00000166794	PPIB	1,856120466
ENSG00000135404	CD63	1,850434249
ENSG00000101310	SEC23B	1,844652995
ENSG00000160058	BSDC1	1,839187521
ENSG00000104412	EMC2	1,83386676
ENSG00000101194	SLC17A9	1,827382982
ENSG00000151779	NBAS	1,827131298
ENSG00000177888	ZBTB41	1,821684876
ENSG00000123562	MORF4L2	1,821253964
ENSG00000128891	CCDC32	1,820637324
ENSG00000154813	DPH3	1,814455126
ENSG00000139182	CLSTN3	1,799666404
ENSG00000133398	MED10	1,791634532
ENSG00000181704	YIPF6	1,791431366
ENSG00000162236	STX5	1,791281987
ENSG00000136868	SLC31A1	1,781123343
ENSG00000112972	HMGCS1	1,772437873
ENSG00000062716	VMP1	1,757327209
ENSG00000142186	SCYL1	1,74541189
ENSG00000144224	UBXN4	1,736639044
ENSG00000111711	GOLT1B	1,731595248
ENSG00000171311	EXOSC1	1,731591505
ENSG00000106400	ZNHIT1	1,725414408
ENSG00000144747	TMF1	1,703513764

ENSG00000121073	SLC35B1	1,701156645
ENSG00000117385	P3H1	1,696798443
ENSG00000154305	MIA3	1,696180196
ENSG00000095139	ARCN1	1,695589531
ENSG00000134375	TIMM17A	1,692417147
ENSG00000128699	ORMDL1	1,692187517
ENSG00000177683	THAP5	1,689718472
ENSG00000148248	SURF4	1,683220995
ENSG00000070495	JMJD6	1,682227427
ENSG00000145740	SLC30A5	1,678435835
ENSG00000144674	GOLGA4	1,666745309
ENSG00000128059	PPAT	1,659793418
ENSG00000162734	PEA15	1,657625798
ENSG00000143515	ATP8B2	1,656975589
ENSG00000051108	HERPUD1	1,655445528
ENSG00000079332	SAR1A	1,649729523
ENSG00000094804	CDC6	1,647713165
ENSG00000197157	SND1	1,645813451
ENSG00000162910	MRPL55	1,639478713
ENSG00000145354	CISD2	1,636824292
ENSG00000167881	SRP68	1,636312768
ENSG00000198862	LTN1	1,630071432
ENSG00000035928	RFC1	1,623453237
ENSG00000082996	RNF13	1,616321431
ENSG00000118705	RPN2	1,61475766
ENSG00000087074	PPP1R15A	1,614035128
ENSG00000179218	CALR	1,613103677
ENSG00000183486	MX2	1,611803237
ENSG00000129484	PARP2	1,610843515
ENSG00000133835	HSD17B4	1,603788439
ENSG00000168404	MLKL	1,603743443
ENSG00000198417	MT1F	1,603058274
ENSG00000109572	CLCN3	1,594295078
ENSG00000100991	TRPC4AP	1,588208653
ENSG00000182325	FBXL6	1,586751815
ENSG00000197930	ERO1A	1,583330192
ENSG00000138069	RAB1A	1,578995911
ENSG00000100412	ACO2	1,570810407
ENSG00000197217	ENTPD4	1,567254437
ENSG00000111540	RAB5B	1,56115532
ENSG00000134851	TMEM165	1,548768568
ENSG00000129317	PUS7L	1,547764766

ENSG00000129083	COPB1	1,547202782
ENSG00000182827	ACBD3	1,54604191
ENSG00000169976	SF3B5	1,544700386
ENSG00000104805	NUCB1	1,542554809
ENSG00000118363	SPCS2	1,542039231
ENSG00000160209	PDXK	1,531564614
ENSG00000014123	UFL1	1,52699038
ENSG00000132842	AP3B1	1,525744661
ENSG00000008952	SEC62	1,525568719
ENSG00000147649	MTDH	1,524516666
ENSG00000174780	SRP72	1,522799304
ENSG00000036054	TBC1D23	1,514558101
ENSG00000168610	STAT3	1,505024222
ENSG00000277443	MARCKS	1,504823656
ENSG00000100938	GMPR2	1,504411471
ENSG00000010270	STARD3NL	1,494913933
ENSG00000140688	C16orf58	1,485774048
ENSG00000164211	STARD4	1,483838837
ENSG00000167842	MIS12	1,476715181
ENSG00000172432	GTPBP2	1,476354583
ENSG00000106348	IMPDH1	1,474184592
ENSG00000072042	RDH11	1,471815435
ENSG00000198466	ZNF587	1,437784802
ENSG00000161204	ABCF3	1,425779914
ENSG00000169223	LMAN2	1,424527661
ENSG00000141756	FKBP10	1,419983447
ENSG00000143621	ILF2	1,411622008
ENSG00000173545	ZNF622	1,411267594
ENSG00000061987	MON2	1,410947872
ENSG00000106153	CHCHD2	1,406344534
ENSG00000078747	ITCH	1,404211154
ENSG00000138674	SEC31A	1,397042144
ENSG00000104969	SGTA	1,394603425
ENSG00000170348	TMED10	1,387371137
ENSG00000172115	CYCS	1,381409088
ENSG00000125651	GTF2F1	1,374100852
ENSG00000173113	TRMT112	1,367436954
ENSG00000084652	TXLNA	1,365843493
ENSG00000121931	LRIF1	1,352876168
ENSG00000149792	MRPL49	1,350865149
ENSG00000112514	CUTA	1,350483311
ENSG00000105355	PLIN3	1,350297324

ENSG00000140105	WARS	1,348991598
ENSG00000168282	MGAT2	1,345800896
ENSG00000159199	ATP5MC1	1,342733779
ENSG00000100994	PYGB	1,33429673
ENSG00000176986	SEC24C	1,324563305
ENSG00000008294	SPAG9	1,323401156
ENSG00000196352	CD55	1,309572661
ENSG00000148606	POLR3A	1,309467542
ENSG00000145725	PPIP5K2	1,30716792
ENSG00000154832	CXXC1	1,292444087
ENSG00000185624	P4HB	1,291497499
ENSG00000116857	TMEM9	1,288444576
ENSG00000281649	EBLN3P	1,276765238
ENSG00000136758	YME1L1	1,268202582
ENSG00000105856	HBP1	1,268034341
ENSG00000065548	ZC3H15	1,261192381
ENSG00000186480	INSIG1	1,258743261
ENSG00000158869	FCER1G	1,256988222
ENSG00000163191	S100A11	1,255017733
ENSG00000198363	ASPH	1,252763913
ENSG00000142227	EMP3	1,243169309
ENSG00000183291	SELENOF	1,242641898
ENSG00000156256	USP16	1,241738326
ENSG00000113282	CLINT1	1,23672813
ENSG00000113716	HMGXB3	1,223407899
ENSG00000001631	KRIT1	1,212814001
ENSG00000143226	FCGR2A	1,193633592
ENSG00000126432	PRDX5	1,161227493
ENSG00000077380	DYNC1I2	1,158449351
ENSG00000114902	SPCS1	1,156687293
ENSG00000110917	MLEC	1,137071732
ENSG00000179632	MAF1	1,131970498
ENSG00000169429	CXCL8	1,130117293
ENSG00000029364	SLC39A9	1,128717862
ENSG00000087086	FTL	1,108145237
ENSG00000025796	SEC63	1,106837828
ENSG00000130164	LDLR	1,105962684
ENSG00000117592	PRDX6	1,104607418
ENSG00000108344	PSMD3	1,102239585
ENSG00000143761	ARF1	1,099249455
ENSG00000135677	GNS	1,093400653
ENSG00000175931	UBE2O	1,086353475

ENSG00000166747	AP1G1	1,071129227
ENSG00000136628	EPRS	1,067973302
ENSG00000143774	GUK1	1,056523905
ENSG00000112977	DAP	1,055664717
ENSG00000090857	PDPR	1,036059756
ENSG00000171241	SHCBP1	1,026098572
ENSG00000143570	SLC39A1	1,01541984
ENSG00000138160	KIF11	-1,020196847
ENSG00000100714	MTHFD1	-1,053516588
ENSG00000178035	IMPDH2	-1,191585061
ENSG00000205268	PDE7A	-1,198238627
ENSG00000146083	RNF44	-1,201639358
ENSG00000134986	NREP	-1,860783371

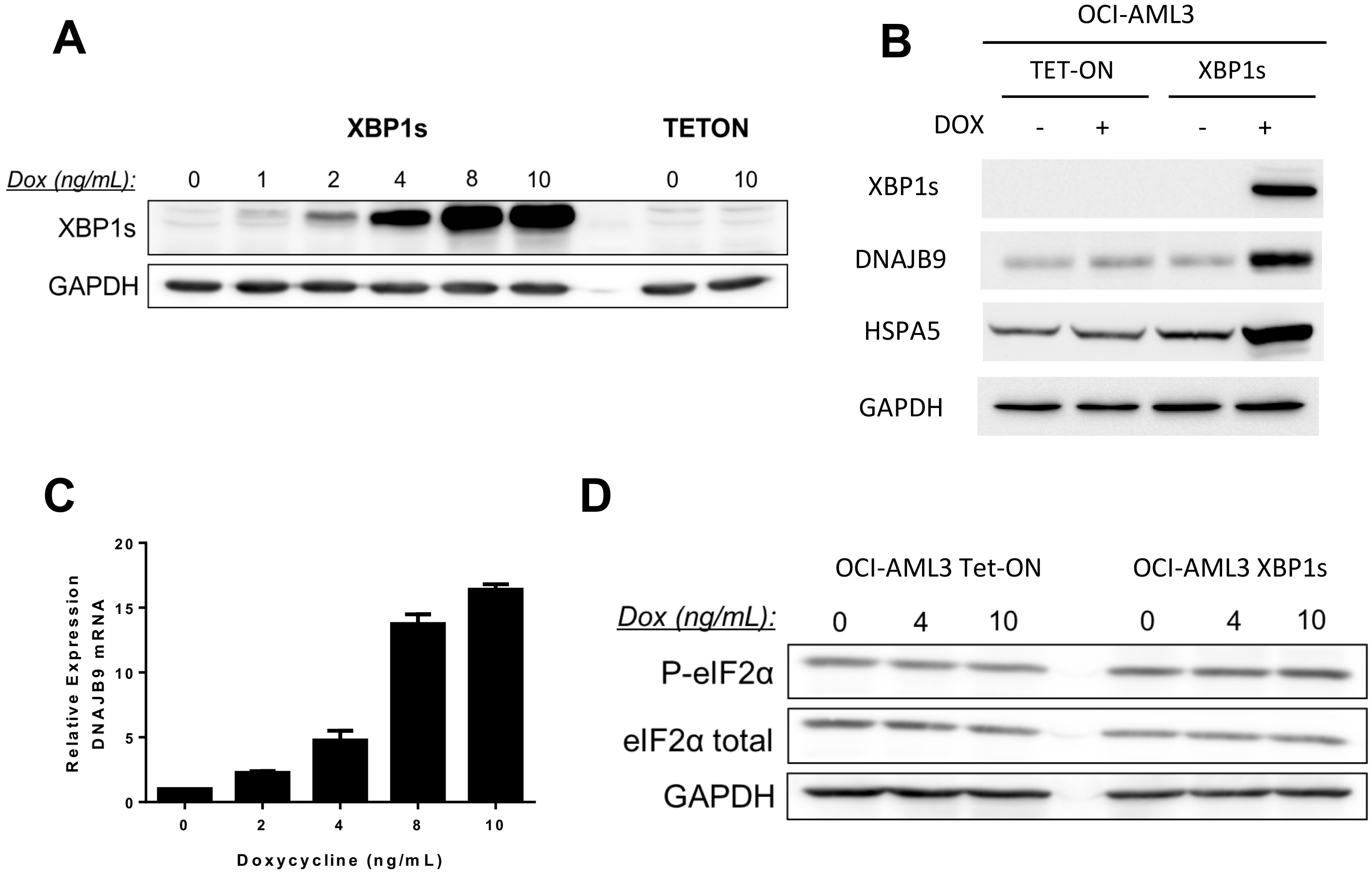

<u>Supplementary Figure 1 :</u> Validation of the inducible expression of XBP1s in OCI-AML3 cell line

(A, B) OCI-AML3 XBP1s-expressing cells and TETON control cells were treated with increasing doses of doxycycline (Dox ; from 0 to 10 ng/mL) for 48H. **(A)** XBP1s protein expression was evaluated by western blotting. GAPDH was used as loading control. **(B)** DNAJB9 and HSPA5 (BiP) protein levels were analyzed by western blotting in OCI-AML3 TET-ON control or XBP1s-expressing cells untreated (-) or treated (+) for 48h with 10ng/ml of doxycycline. GAPDH is used as loading control. **(C)** DNAJB9 mRNA levels were determined by RT-qPCR. Expression values were normalized to the housekeeping gene ABL, and are depicted as a ratio of expression in doxycycline-treated cells relative to untreated cells **(D)** OCI-AML3 XBP1s-expressing cells and TETON control cells were treated with increasing doses of doxycycline (Dox ; from 0 to 10 ng/mL) for 48H. eIF2α phosphorylation (P-eIF2α) and eIF2α total protein expression were evaluated by western blotting . GAPDH was used as loading control.

A

B

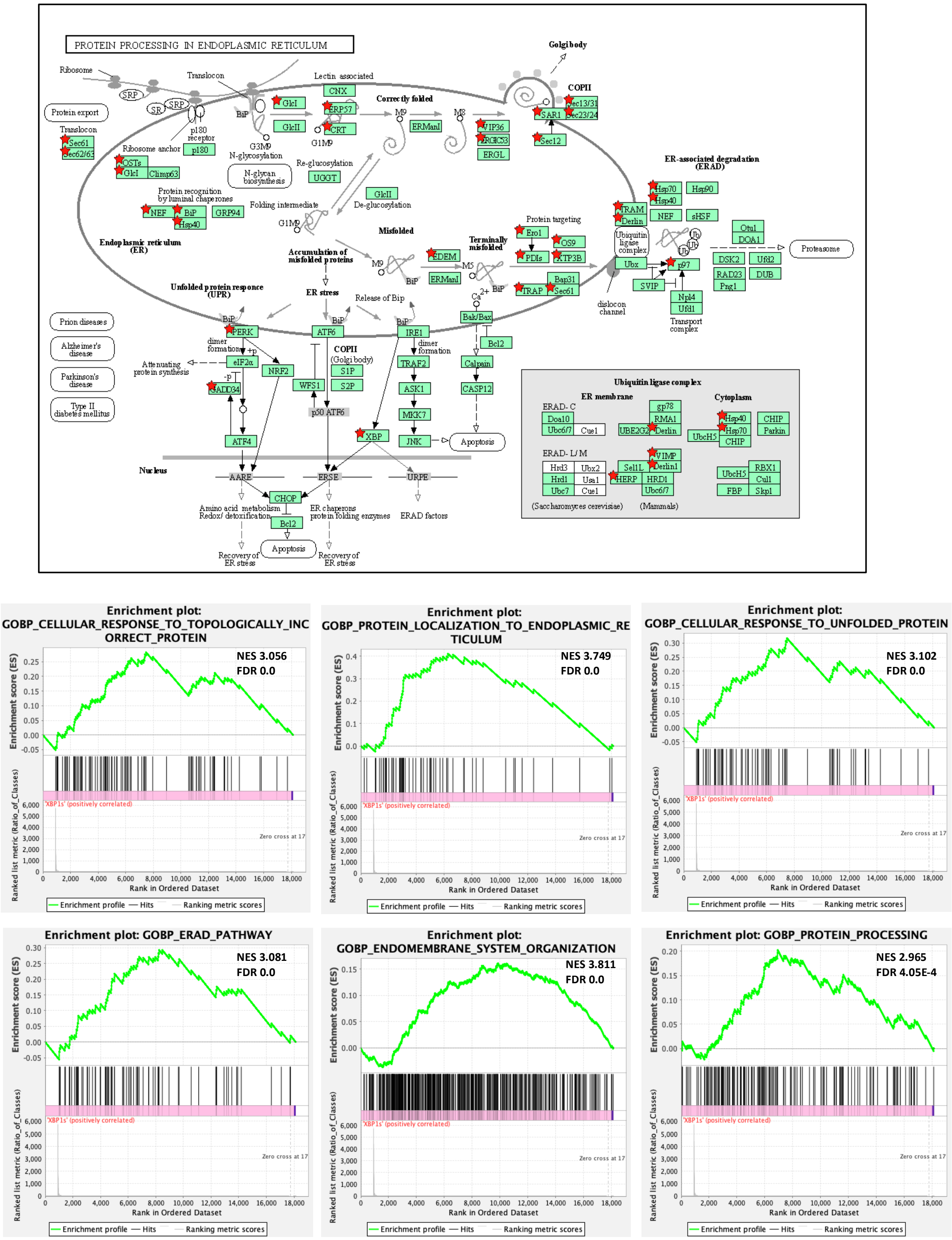

***Supplementary Figure 2*:**

(A) Among the 390 selected genes overlapping between RNASeq and ChIP seq experiments (*i.e.,* direct XBP1s targets candidates) many genes (labeled here with a red star) code for proteins involved in protein processing in the endoplasmic reticulum. Pathway map image generated by KEGG (Kanehisa, M. and Goto, S.; KEGG: Kyoto Encyclopedia of Genes and Genomes. (2000) Nucleic Acids Res. 28, 27-30).

(B) Gene set enrichment analysis (GSEA) performed on the RNASeq full data of OCI-AML3-XBP1s cells treated with doxycycline. NES, normalized enrichment score. FDR, false discovery rate

Supplementary Figure 3

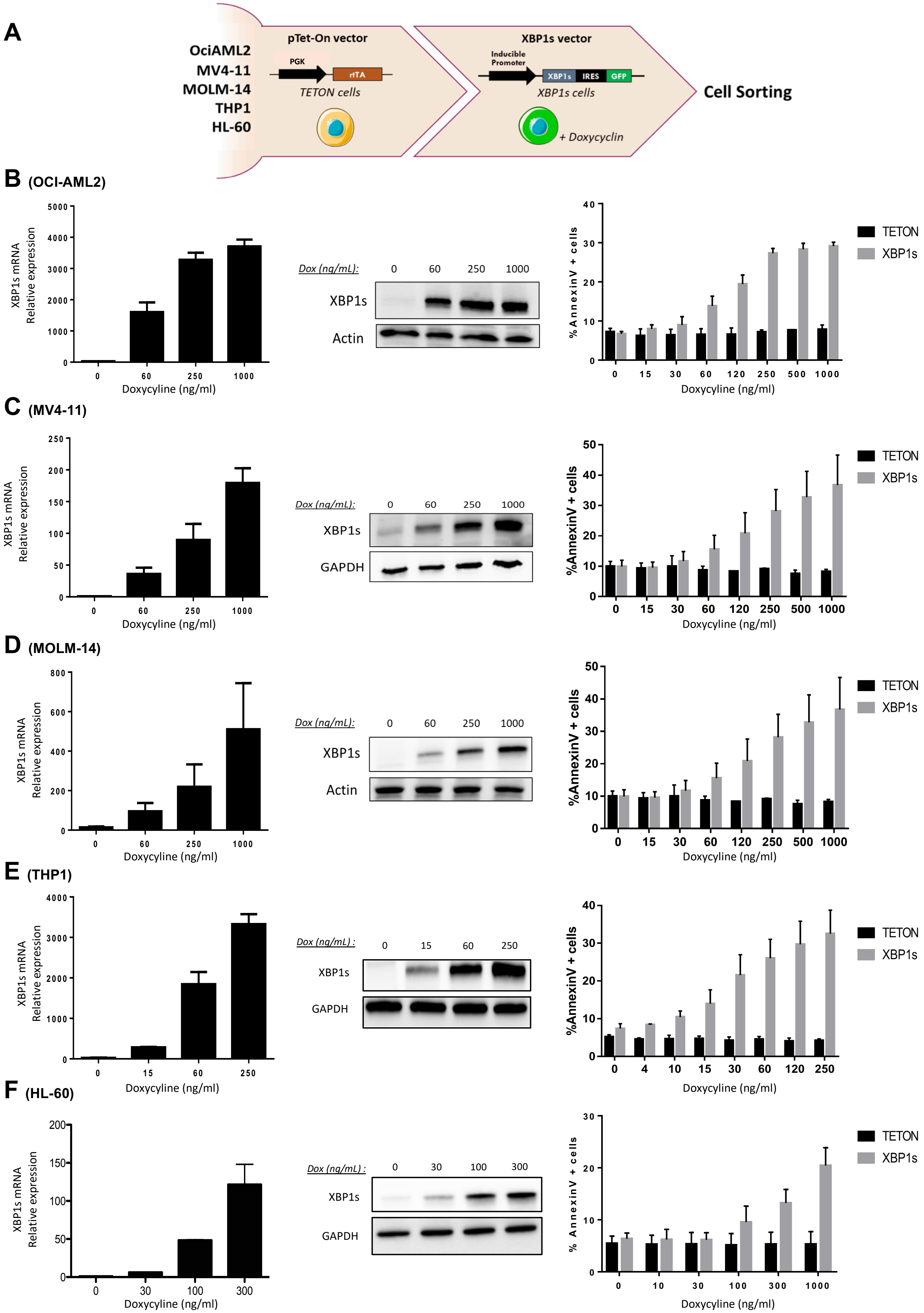

Supplementary Figure 3: XBP1s expression induces apoptosis in various representative AML cell lines in vitro

(A) Schematic of XBP1s-inducible model generation: In the same way as for OCI-AML3 cells, the 5 different AML cell lines were first transduced by a lentivector expressing the rtTA transgene. The obtained "Tet-On " cells were then transduced a second time with a lentivector expressing the XBP1s transgene followed by an IRES-eGFP cassette under the control of a TET inducible promoter. Inducible, GFP positive cells were sorted using flow cytometry after 24h of doxycycline treatment.
(B) OCI-AML-2, **(C)** MV4-11, **(D)** MOLM-14, **(E)** THP1, and **(F)** HL60 cells were treated with increasing amounts of doxycycline during 48h.
Left panels: *XBP1s* expression was evaluated by RT-qPCR. Expression values were normalized to the housekeeping genes *HPRT, MLN51* and *ABL*, and are depicted as a ratio of mRNA expression in doxycycline-treated cells relative to untreated cells.
Middle panels: XBP1s protein level was evaluated by Western Blot; Actin or GAPDH were used as loading controls.
Right panels: Apoptosis was measured by flow cytometry using Annexin V/PI staining. Data represent mean ± SD (n=3).

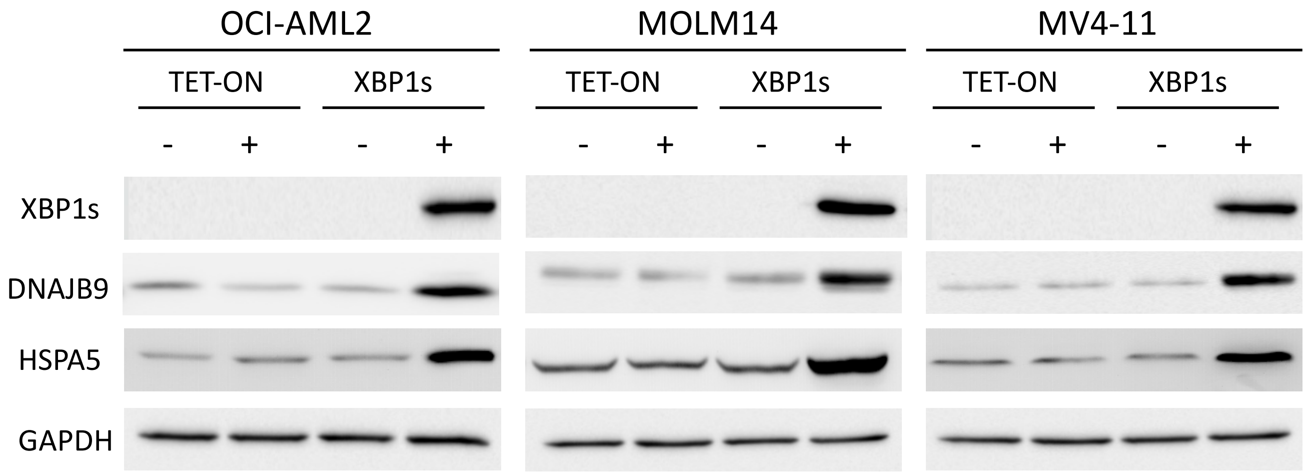

***Supplementary Figure 4*: Validation of the XBP1s inducible expression in AML cell lines**

DNAJB9 and HSPA5 (BiP) protein levels were analyzed by western blotting in OCI-AML2, MOLM-14 and MV4-11 TET-ON control or XBP1s-expressing cells untreated (-) or treated (+) for 24h with 100ng/ml of doxycycline. GAPDH was used as loading control.

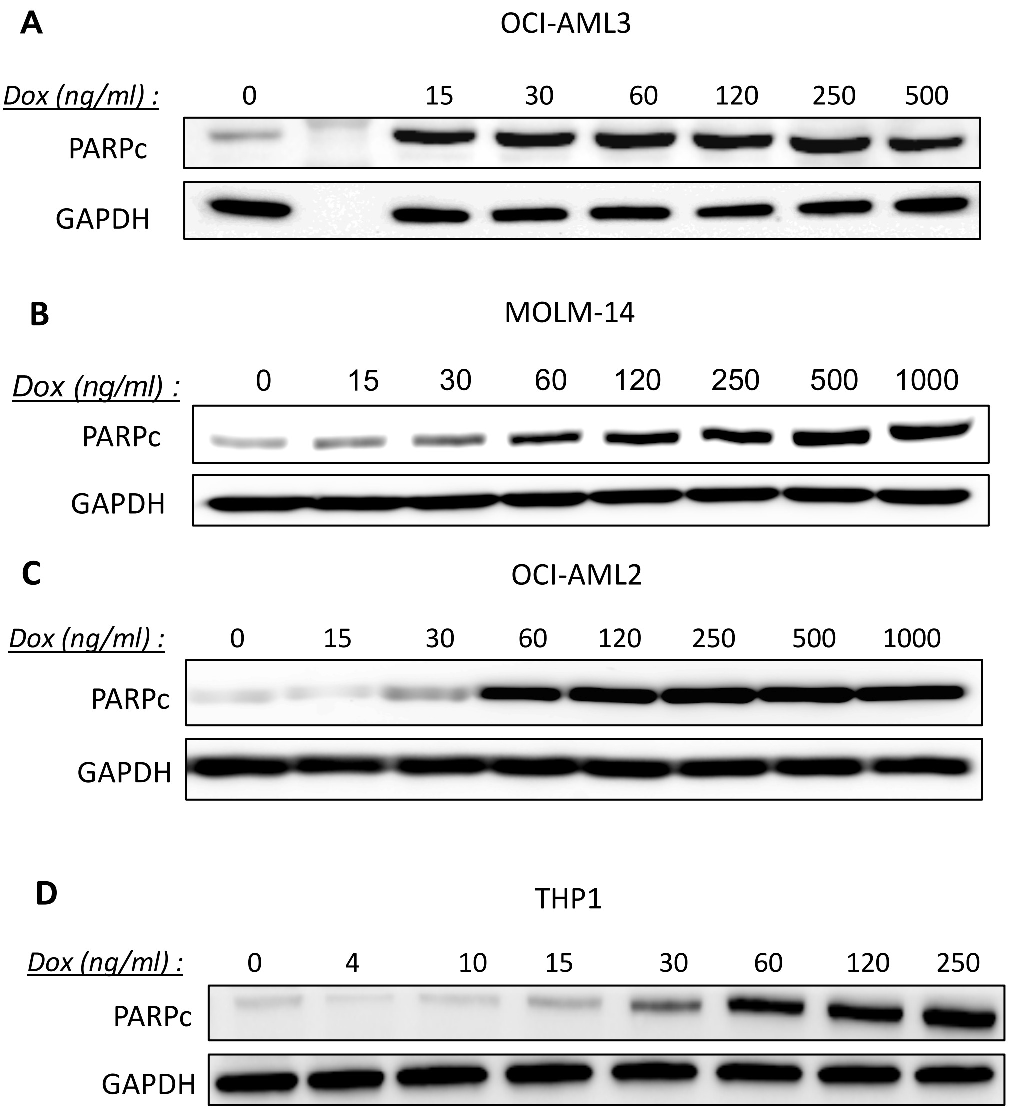

Supplementary Figure 5**: XBP1s induces PARP cleavage in AML cell lines**

(A-D) OCI-AML3, MOLM-14, OCI-AML2 and THP1 XBP1s-expressing cells were treated with increasing amounts of doxycycline during 48h. Cleaved PARP (PARPc) protein level was evaluated by Western Blot; GAPDH was used as loading control.

A **miRNA sequencing analyses**

miRNA	Fold Change
hsa-miR-22-3p	18,7882022
hsa-miR-199a-3p	17,125
hsa-miR-148a-5p	10,60199
hsa-miR-148a-3p	9,91198555
hsa-miR-21-3p	6,56037515
hsa-miR-574-3p	6,0952381
hsa-miR-215-5p	5,5
hsa-miR-29c-3p	3,78571429
hsa-miR-1307-5p	2,95053004
hsa-miR-501-3p	2,875
hsa-miR-21-5p	2,73601533
hsa-miR-877-5p	2,7

B **RNA sequencing ChIP Sequencing analyses**

Ensembl Reference	Gene Symbol	Fold Change
ENSG00000257605	AC073611.1	145,5190418
ENSG00000272405	AL365181.3	104,1263083
ENSG00000248323	LUCAT1	65,25434864
ENSG00000233452	STXBP5-AS1	62,71774624
ENSG00000236453	AC003092.1	60,14882097
ENSG00000186594	MIR22HG	42,43514688
ENSG00000259343	TMC3-AS1	24,90193321
ENSG00000281649	EBLN3P	2,42295102
ENSG00000227706	AL713998.1	0,53981096
ENSG00000274536	AL034397.3	0,42778662
ENSG00000247982	LINC00926	0,39116138
ENSG00000215246	AC116351.1	0,32615199
ENSG00000269243	AC008894.2	0,23990873

***Supplementary Figure 6*: microRNA sequencing and ChIPseq analyses**

(A) Micro-RNAs upregulated more than 2-fold upon XBP1s expression in OCI-AML3 XBP1s cells compared to Tet-On control cells after treatment with 10ng/mL of doxycycline for 48H.
(B) Direct XBP1s long non-coding RNAs target genes identified as enriched in ChiP sequencing and regulated more than 2-fold upon XBP1s expression in OCI-AML3 XBP1s cells compared to Tet-On control cells after treatment with 10ng/mL doxycycline for 48H.

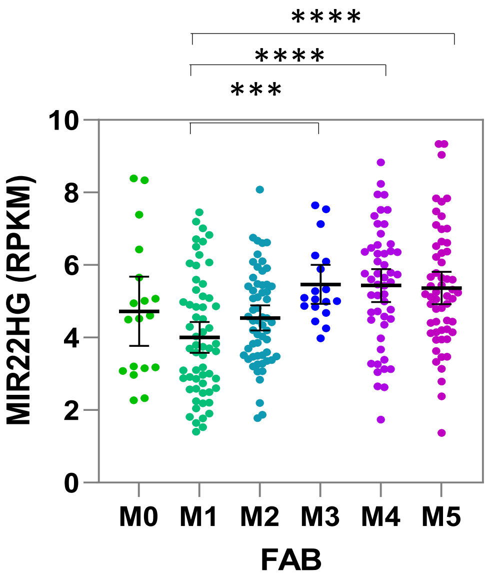

Supplementary Figure 7: **Expression of MIR22HG transcript in AML patient samples**
Each dot shows expression of MIR-22HG in AML FAB categories of the Beat-AML cohort (Beat-AML diagnosis samples for which the FAB is known - n = 275).
X axes: M0 = undifferentiated; M1 = myeloblastic without maturation; M2 = myeloblastic with maturation; M3 = promyelocytic; M4 = myelomonocytic; M5 = monocytic;
Unpaired Student t test (***: $p < 0.005$, ****: $p < 0.001$) was used for sample inter-group significance.

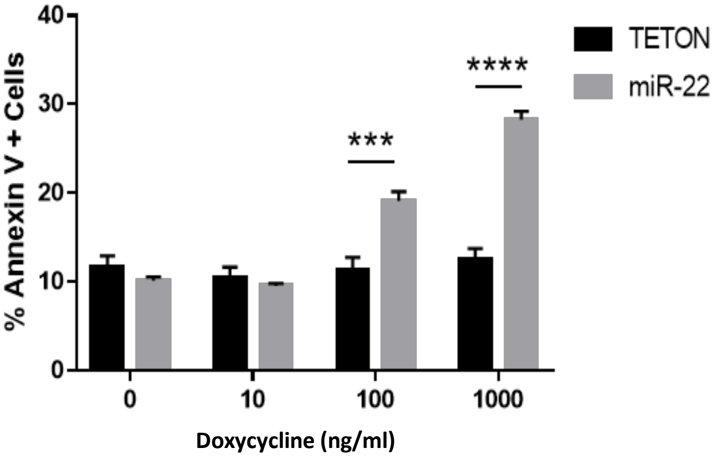

***Supplementary Figure 8*: Doxycycline-inducible miR-22 expression induces apoptosis in OCI-AML2- cells**

OCI-AML2 Tet-On cells were transduced with a lentivector expressing a dox-inducible miR-22 construct. Transduced cells and Tet-On (control) cells were then treated with increasing amounts of doxycycline during 48h. Apoptosis was measured by flow cytometry using Annexin V/PI staining. Data represent mean ± SD (n=3). Statistical analysis was performed using unpaired *t*-tests (***p≤0.001, ****p ≤ 0,0001).

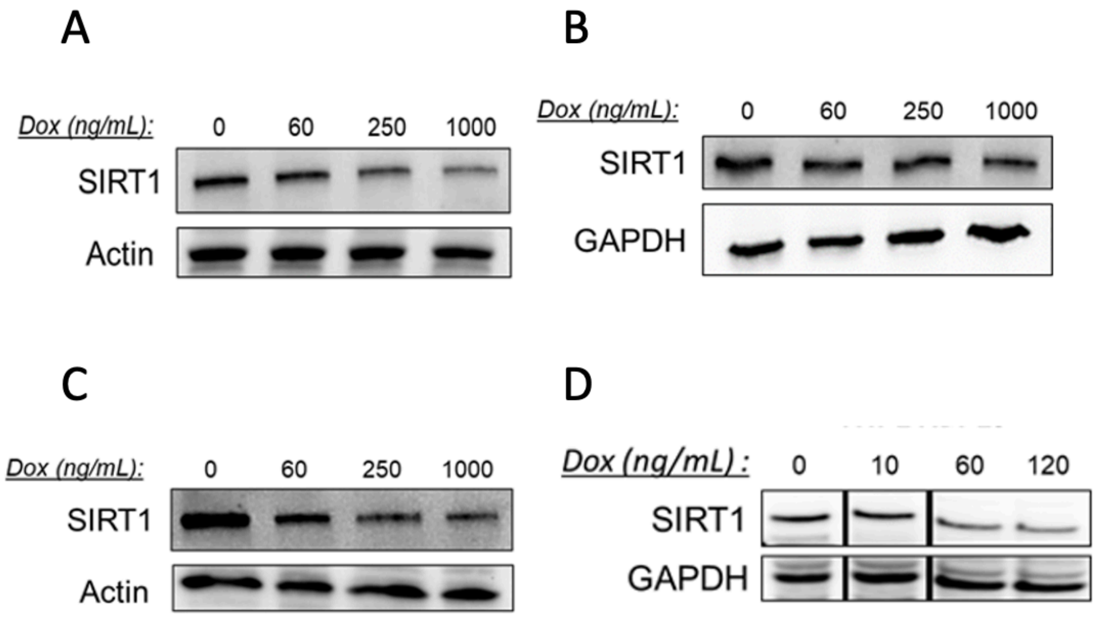

Supplementary Figure 9: **XBP1s expression induces a downregulation of SIRT1 protein expression**

(A-D) SIRT1 protein levels were analyzed by western blotting in MOLM-14 (A), MV4-11 (B), OCI-AML2 (C) and THP1 (D) XBP1s-expressing cells after treatment with the indicated concentrations of doxycycline.

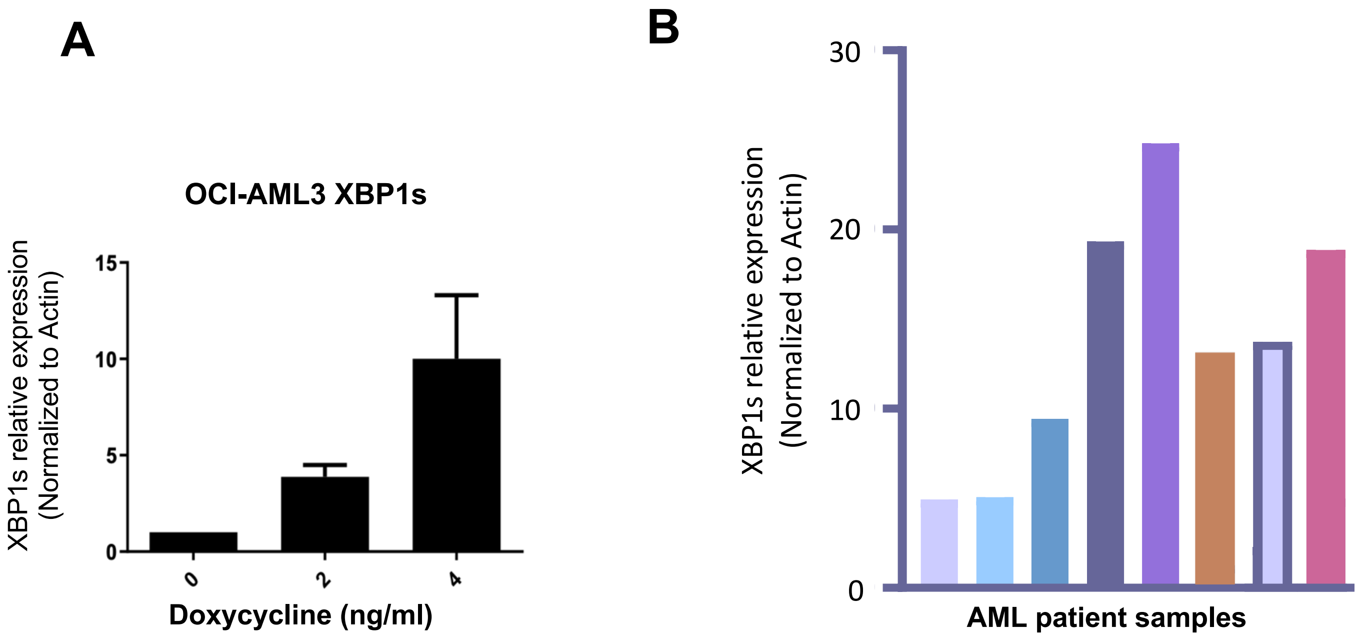

Supplementary Figure 10: XBP1s induction level in the OCI-AML3 XBP1s cell line and in patient samples treated with tunicamycin

(A) OCI-AML3 XBP1s expressing cells were treated with 2 and 4 ng/ml of doxycycline for 48h. *XBP1s* expression was evaluated by RT-qPCR. Expression values were normalized to actin, and are depicted as a ratio of mRNA expression in doxycycline-treated cells relative to untreated cells.

(B) AML patient samples were treated or not for 4 hours with 1µg/mL of tunicamycin. *XBP1s* expression was evaluated by RT-qPCR. Expression values were normalized to actin, and are depicted as a ratio of mRNA expression in tunicamycin -treated patient cells relative to their untreated counterpart.
